# A Review of Hybrid Manufacturing: Integrating Subtractive and Additive Manufacturing

**DOI:** 10.3390/ma18184249

**Published:** 2025-09-10

**Authors:** Bruno Freitas, Vipin Richhariya, Mariana Silva, António Vaz, Sérgio F. Lopes, Óscar Carvalho

**Affiliations:** 1 Center for MicroElectroMechanical Systems (CMEMS-Uminho), University of Minho, 4800-058 Guimarães, Portugal; vipinrichhariya7@gmail.com (V.R.); m.silva@dem.uminho.pt (M.S.); oscar.carvalho@dem.uminho.pt (Ó.C.); 2 Algoritmi Research Centre, University of Minho, 4800-058 Guimarães, Portugal; aivaz@dps.uminho.pt (A.V.); sergio.lopes@dei.uminho.pt (S.F.L.)

**Keywords:** additive manufacturing, hybrid manufacturing, multimaterial, robotics, toolpath

## Abstract

It is challenging to manufacture complex and intricate shapes and geometries with desired surface characteristics using a single manufacturing process. Parts often need to undergo post-processing and must be transported from one machine into another between steps. This makes the whole process cumbersome, time-consuming, and inaccurate. These shortcomings play a major role during the manufacturing of micro and nano products. Hybrid manufacturing (HM) has emerged as a favorable solution for these issues. It is a flexible process that combines two or more manufacturing processes, such as additive manufacturing (AM) and subtractive manufacturing (SM), into a single setup. HM works synergistically to produce complex, composite, and customized components. It makes the process more time efficient and accurate and can prevent unnecessary transportation of parts. There are still challenges ahead regarding implementing and integrating sensors that allow the machine to detect defects and repair or customize parts according to needs. Even though modern hybrid machines forecast an exciting future in the manufacturing world, they still lack features such as real-time adaptive manufacturing based on sensors and artificial intelligence (AI). Earlier reviews do not profoundly elaborate on the types of laser HM machines available. Laser technology resolutely handles additive and subtractive manufacturing and is capable of producing groundbreaking parts using a wide scope of materials. This review focuses on HM and presents a compendious overview of the types of hybrid machines and setups used in the scientific community and industry. The study is unique in the sense that it covers different HM setups based on machine axes, materials, and processing parameters. We hope this study proves helpful to process, plan, and impart productivity to HM processes for the betterment of material utilization and efficiency.

## 1. Introduction—Origins of Hybrid Manufacturing

From pigments in cave art to the Gutenberg press of the 15th century, and later to our home inkjet printers, we have finally arrived at revolutionary three-dimensional (3D) printers [1,2]. It was not until the 1980s, thanks to pioneers like Charles Hull, that we left the two-dimensional realm and jumped to the third dimension with the development of stereolithography (SLA), one of the mainstays of contemporary 3D printing [2].

The existence of a diverse array of manufacturing processes offers viable solutions to the modern manufacturing industry; however, it also makes identifying an optimal option more challenging. The advent of additive manufacturing (AM), along with computer three-dimensional (3D) models, made it possible to create highly detailed and intricate parts. AM is one process that generates complex parts by depositing and stacking material layer by layer [3]. On the other hand, subtractive manufacturing (SM) removes excess material from a part and can achieve a flawless surface finish. The production of intricate parts at the micro and nano levels requires utmost precision and several forms of subtraction and addition of material layers. Combining additive manufacturing with subtractive manufacturing represents a feasible approach, as demonstrated by hybrid systems that integrate metal Powder Bed Fusion using a high-power ytterbium fiber laser with subsequent high-speed CNC milling on the same build platform [3]. This combination leverages additive manufacturing’s ability to produce complex geometries and internal features, alongside subtractive methods that ensure tight tolerances and superior surface finish. Such synergy opens a new paradigm of possibilities, known as hybrid manufacturing (HM).

### 1.1. Additive Manufacturing (AM)

**Figure 1:**
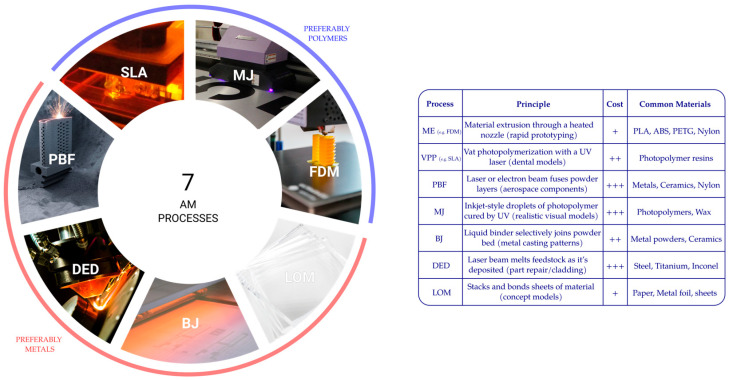
Exploring the seven processes of additive manufacturing. Material Extrusion (FDM and FFF), Vat Photopolymerization (SLA and DLP), Powder Bed Fusion (PBF, SLS, DMLS, and SLM), Material Jetting (MJ), Binder Jetting (BJ), Directed Energy Deposition (DED), and Sheet Lamination (LOM). Cost Legend: +—Entry-level to mid-range desktop systems & low material cost; ++—Professional to small industrial systems & moderate material cost; +++—Industrial machines & high-cost materials/post-processing. (Photographs of SLA, MJ, FDM, LOM, BJ, DED, and PBF, respectively, adapted from Refs. [7,8,9,10,11,12,13]).

The last two decades brought forward AM, which produces parts layer by layer based on a computer mode [3]. Common AM processes include Fused Filament Fabrication (FFF) and stereolithography (SLA or SL). FFF utilizes fused filament, traditionally filament derived from polymers such as Acrylonitrile Butadiene Styrene (ABS) and Polylactic Acid (PLA) to manufacture interesting 3D parts. SLA is a 3D printing technology that uses photopolymerization to create a 3D solid using ultraviolet (UV) light to solidify regions of a liquid photopolymer (resin) that rests inside a container [4]. Incidentally, both FFF and SLA are optimized to solely produce polymeric parts. Another solution is to use laser technologies, such as Powder Bed Fusion (PBF) and Directed Energy Deposition (DED). These two methods provide access to a vast selection of materials, including steel, titanium alloys, thermoplastics, ceramics, etc. [3]. Figure 1 depicts the seven major additive manufacturing processes defined by ISO/ASTM 52900:2015 [5] as Material Extrusion (ME, e.g., FDM), Material Jetting (MJ), Binder Jetting (BJ), Powder Bed Fusion (PBF), Sheet Lamination (SL or LOM), Directed Energy Deposition (DED), and Vat Photopolymerization (VP, e.g., SLA).

**Figure 2:**
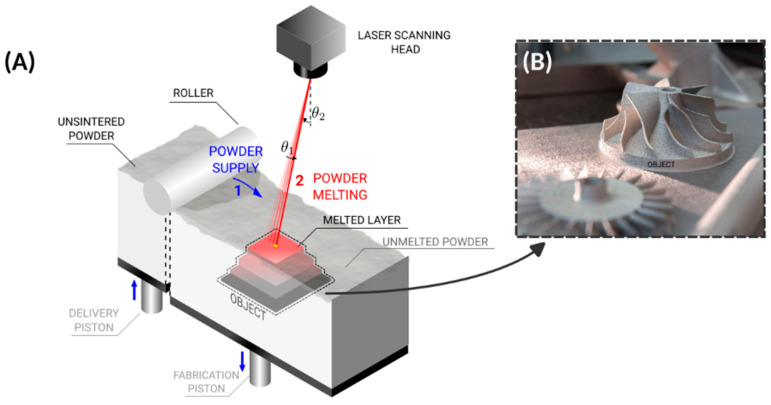
Selective laser melting (SLM). (**A**) Step 1—Unsintered powder is spread with a roller for initial layer preparation and compacting. Step 2—The laser head precisely scans the powder, melting it and creating a layer. The fabrication piston moves down after scanning. The steps are similar for SLS. (**B**) The final object after brushing away excess powder which did not melt. (Photograph adapted from Ref. [15]).

DED and PBF are two of the most cherished HM technologies. PBF includes selective laser sintering (SLS), selective laser melting (SLM) and electron beam melting (EBM). The former two technologies (SLS and SLM) are inherent in what is known as “Laser PBF” (LPBF). In an SLS process, a Computer-Aided Design (CAD) model of the part is first virtually sliced, and several cross-sections are obtained. The SLS machine contains a sink (bed) where metal powder is spread, and a laser beam scans and materializes the first layer of the part. The laser only scans the cross-section resulting from the slicing of the virtual model. Thus, only the powder particles that belong to that area are sintered or melted in the case of SLM, as shown in Figure 2. Subsequently, a second layer of powder is spread on top of the previous one, and the laser produces the next cross-section. In this step, the laser not only materializes the new cross-section but also bonds it to the previous cross-section (in the bottom). This occurs since the layers are thin and close to each other; therefore, heat transfer will naturally fuse subjacent particles, providing a three-dimensional interlayer bond. This process goes on in a layer-by-layer fashion until the final part is entirely produced [6].

**Figure 3:**
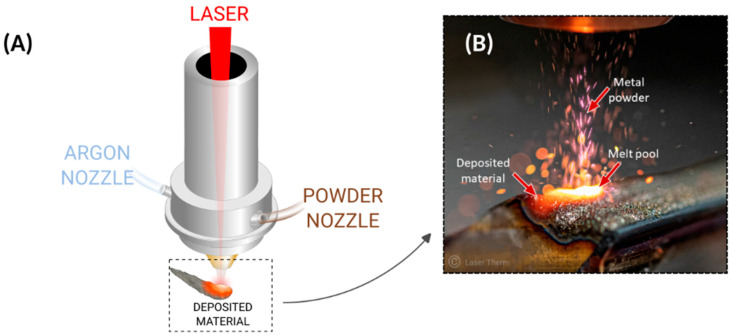
Directed Energy Deposition. (**A**) The powder is deposited and melts due to the laser source. (**B**) A close up of the laser metal deposition process (photograph adapted from Ref. [16]).

DED is a generic term for a 3D printing technology that uses an energy source (usually a laser) to deposit a material, e.g., metal powder (or wire), onto a surface, as shown in Figure 3. Other related names include Laser Powder Cladding (LPC), Laser Engineered Net-Shaping (LENS), Extreme High-Speed Laser Application (EHLA), Laser Direct Metal Deposition (LDMD, DMD, LMD, or DLD), Wire and Arc Additive Manufacturing (WAAM), and fusion using electric arc. DED has the capacity to produce fully dense and gradient parts using spherical powder particles within the range of 50–200 μm. These particles should be melted using a CO_2_ laser if one wishes to deposit thick layers of several millimeters. In contrast, Nd-YAG (neodymium-doped yttrium aluminum garnet) laser is an adequate option for depositing thin layers (less than one millimeter) with very high precision [14].

### 1.2. Subtractive Manufacturing (SM)

**Figure 4:**
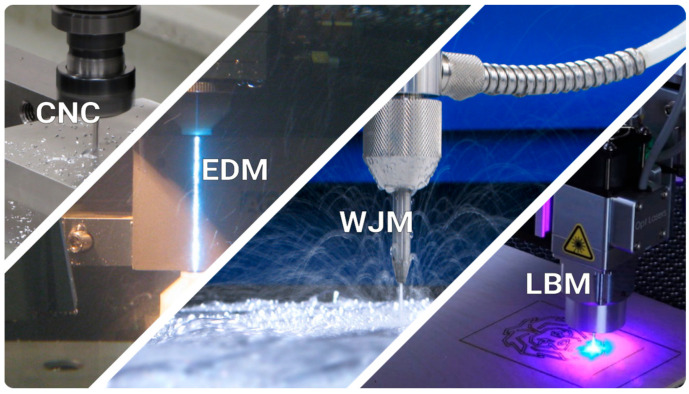
Subtractive processes, including CNC machining, Electrical Discharge Machining (EDM), Water Jet Machining (WJM), and Laser Beam Machining (LBM). (From left to right, photographs adapted from Refs. [20,21,22,23]).

**Figure 5:**
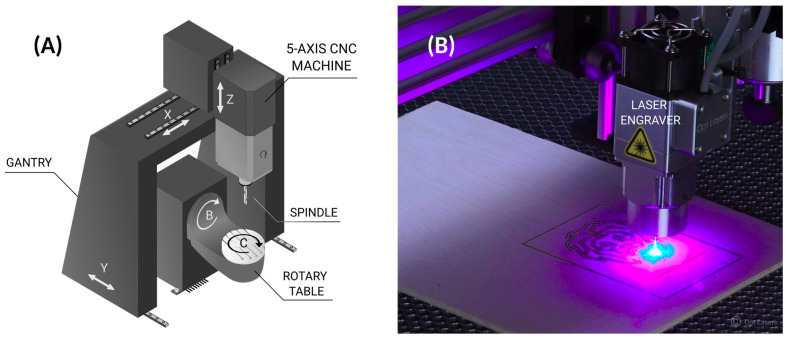
(**A**) Five-axis CNC machine showing its five axes: linear X, Y, Z and rotary B, C. The CNC head contains a spindle that rotates the end mill. (**B**) Instead of a spindle, a laser head can be used to machine, e.g., a board, as shown in the photograph. (Photograph adapted from Ref. [23]).

Subtractive manufacturing is the removal of material from a part either manually or using instructions provided by computer-generated models. Machining techniques such as milling, turning, or drilling are all subtractive by nature. One can broadly categorize subtractive manufacturing processes into CNC machining, Electrical Discharge Machining (EDM), Water Jet Machining (WJM), and Laser Beam Machining (LBM), as shown in Figure 4 [17]. In the 21st century, Computer Numerical Control (CNC) machining gained popularity and is now embedded in emerging manufacturing fields, especially in hybrid machines. However, Numerical Control (NC) machining originates from John Parsons who, in the early 1950s, was motivated to improve how helicopter rotors were manufactured [18]. CNC machining (Figure 5A) is the most widely used subtractive method; however, one should also consider other attractive technologies, such as EDM for intricate features and laser ablation (LA) for microtextures [19].

### 1.3. Hybrid Manufacturing (HM)

**Figure 6:**
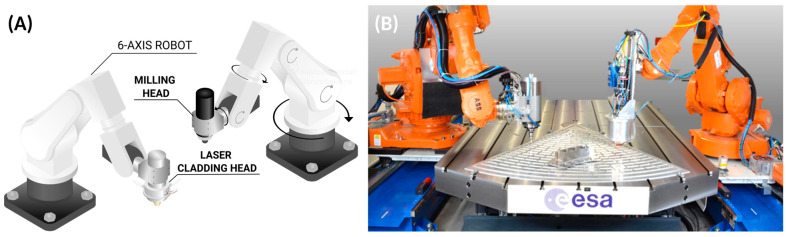
(**A**) Hybrid manufacturing with two 6-axis robots, with one performing milling and the other performing laser metal deposition. (**B**) Photograph of two 6-axis robots working synergistically to produce aerospace grade parts. One robot uses cryogenic milling, and the other uses laser metal deposition. (Photograph adapted from Ref. [25]).

HM combines two or more distinct manufacturing processes, typically additive and subtractive, within a single platform, enabling the creation of complex parts through the combination of material deposition and precision machining without the need to reposition the workpiece [24]. This seamless integration not only streamlines production workflows but also unlocks the design freedom of additive techniques alongside the tight tolerances and surface quality of subtractive methods. Figure 6 illustrates this impressive hybrid technology based on two cooperative robotic arms that work in unison to develop revolutionary parts [25].

**Figure 7:**
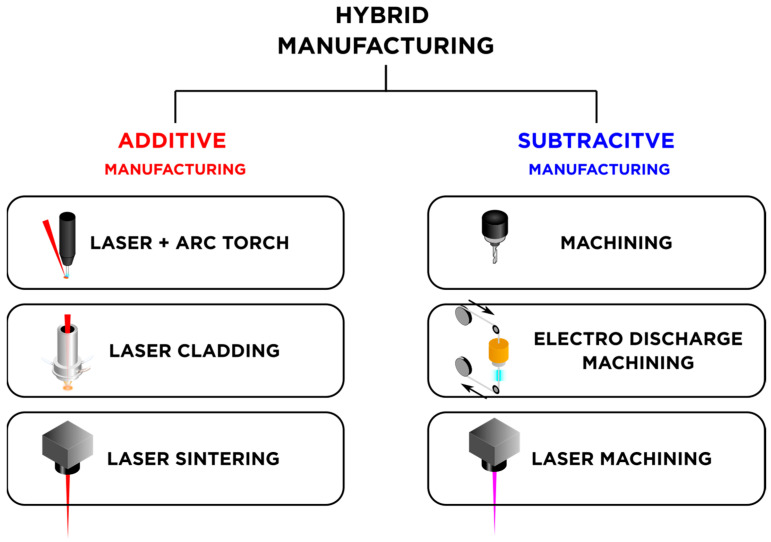
Some AM and SM processes that can be used in a laser hybrid manufacturing machine [24].

A hybrid machine allows the designer to modify existing parts manufactured by the conventional, rapid, and large-scale processes such as die casting. Customization as per the users’ needs is the major advantage of HM. It has a broad scope in the medical industry, being used for patient-specific hip and knee implants, multi-material crowns/veneers, scaffolds, and parts with cell-targeted microstructures. Hybrid parts including multi-material, high-performance, and fiber-reinforced parts are also decisive for automotive, aviation, and aerospace industries. Hence, hybrid technology is a step forward in shaping manufacturing and component repair [26]. Metal and laser-based approaches are typically popular in the world of HM, and Figure 7 shows some examples of hybrid laser manufacturing.

HM is a type of technology that offers a combination of different manufacturing techniques. One such combination is EDM (subtractive manufacturing) with SLM (additive manufacturing), which is an effective approach to print micro metallic patterns on a pre-finished substrate to be used as a microstructure mold [27]. DED (or LMD) is an alternative process to SLM, which can be combined with machining to handle samples such as Inconel 718 (a nickel-chromium alloy) and produce high-temperature metallic structures [28,29].

Three-dimensional dispenser printing is another AM process that can partake in HM. It is a fruitful technology for developing electrical circuits in parts by dispensing either conductive or ceramic pastes. Combined with laser machining, it can fabricate microwave circuits (or antennas) [30]. As expected, integrating electronics with conventional mechanical parts is an exciting way to produce smart components that interact with the environment by sensing or providing stimulus (e.g., piezoelectric actuation).

Laser DED, a layer-wise process, permits the insertion of sensors between layers, thus creating innovative components such as smart tensile bars. A strain gauge sensor may be incorporated in a layer using screen printing. This technology uses inks to build the strain gauge, in particular ceramic ink for insulation, silver particles for the conductor, and platinum particles for the resistor [31]. The integration of sensors into the hybrid manufacturing process itself is a leap forward in terms of part validation, reproducibility, and automation. For instance, an Eddy current detector (probe) can be integrated into the additive/subtractive hybrid manufacturing (ASHM) process in order to inspect a part for internal defects. After detection, repair operations are conducted through milling and then material deposition [29]. Pursuing this line of thought, researchers from Missouri included a stereo vision camera in their hybrid machine to detect defects in the component. Afterwards, a laser displacement sensor 3D scans the defect and proceeds to repair the component based on that scan [30].

Milling efficiency in SM can be improved using several approaches. One such example is the possible reduction in milling forces experienced when processing titanium alloy workpieces (Ti-6AL-4V). The experiment consists of a KUKA robot with a 2.5 kW Nd:YAG laser for LMD. The additive manufactured titanium piece (Ti-6Al-4V) is placed in a CNC machine that contains a heating device, and a reduction in milling forces is observed when the workpiece temperature is greater than 300 °C due to the thermal softening effect [31]. Laser-assisted machining (LAM) is another prominent subtracting method where the laser, in some circumstances, can reduce the cutting force by more than 40% due to laser preheating [32]. On the other hand, cryogenic milling can also be used for hard-to-cut materials, including the aforementioned Ti-6AL-4V. This results in clean, residue-free surfaces compared to dry or cooling lubricant machining [25].

HM produces versatile parts and is not restricted to monolithic and mono-material components. Fabricating injection molding inserts with conformal cooling is one of many signs of its abilities. These inserts can be fabricated using SLM and traditional milling using the following materials: maraging steel as powder for the SLM-processed parts; C5 steel; and high-conductivity copper alloy (Ampcoloy 83) for milled parts [33]. Moreover, researchers have used LENS and Wire EDM to fabricate titanium–titanium diboride (Ti-TiB2) composites [34]. In the biomedical field, dental implants are also components that can greatly benefit from being designed as multi-material or hybrid components. Zirconia implants can be textured by first machining tracks with a Nd:YAG laser, then depositing hydroxyapatite (Hap) powder onto such tracks, and finally sintering the powder with a CO_2_ laser (additive process) [35,36]. Another biomedical application is the fabrication of titanium endosseous implants with optimized surfaces for enhancing osteogenic differentiation of human mesenchymal stem cells. This can be achieved by manufacturing titanium alloy (Ti6Al4V) specimens using SLM and further modifying them using femtosecond laser (FS) ablation (subtractive step) [37].

Integrated HM machines that operate continuously can reduce the production time of complex parts [26]; for example, DMG Lasertec 65 integrates a five-axis coaxial nozzle (DED) with traditional machining (DMG MORI—Iga, Mie, Japan) [38]. The Mazak VC-500A/5x AM is another powerful and integrated hybrid machine that can fabricate a component using 316 L stainless steel wire. This additive process is marketed as hot-wire deposition (HWD) (Yamazaki Mazak Corporation—Florence, KY, USA). Using such hybrid machine allows the overall cycle time to be reduced by 68% [39]. Finer resolution hybrid machines support the creation of 3D structures in soft materials, including channels, overhangs, and undercuts with a minimum resolution of about 3 μm. This is the case of Hybrid Laser Printing (HLP) based on femtosecond laser (FS laser) (Soman Lab, Syracuse University—Syracuse, NY, USA) [40].

A more exotic hybrid process is the combination of the additive LMD process and the subtractive Jet Electrochemical Machining (JECM or Jet-ECM) process into a single hybrid technology named LMD-JECM. The setup consists of a six-axis KUKA robot (Augsburg, Germany) that contains an LMD head, which can be switched to a JECM head that contains a machining cathode, a soft brush, and a grinding tool (hard brush) [41]. Another interesting hybrid setup is the utilization of a special coaxial nozzle with shielding gas (helium) to produce large titanium (Ti-6Al-4V) components, graded for use in the (ATHENA) telescope (Fraunhofer IWS—Dresden, Germany) [42].

While there are elegant and comprehensive reviews about hybrid manufacturing in the literature, these do not provide an in-depth exploration of the engineering behind laser hybrid machines which dominate the market. Moreover, the intricate details of each type of machine are not analyzed and compared across existing studies. There is a need to provide a detailed overview of existing hybrid machines and setups used by the scientific community and industry. This review gathers scientific studies from the literature on laser hybrid machines. It further describes modern studies that help pave the way to new developments in the area of HM. Furthermore, we provide panoramic and technical analyses of each hybrid machine, its utilization, materials used, and the respective processing parameters.

## 2. Methodology—Literature Search

Besides the traditional narrative review, we also conducted a systematic analysis to obtain a bird’s-eye view of the leading manufacturing processes, techniques, and materials. The present review intends to answer the following question: what are the state-of-the-art hybrid manufacturing machines, and how are their operating principles classified? To answer this question a literature search was performed using prominent scientific databases.

**Figure 8:**
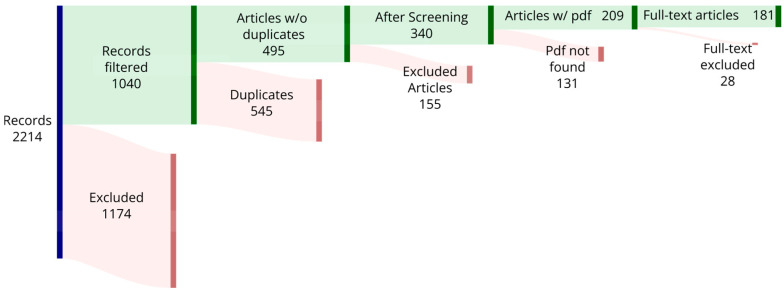
Sankey diagram. Red: excluded articles; green: included articles.

The discussed articles are no more than 7 years old, ranging from 2018 to 2025; in particular, the systematic analysis focuses on studies up to 2023, and additional studies up to 2025 are considered in the discussion to reflect the most recent developments. The database search included articles from Doaj, Scopus, Web of Science, and PubMed using the key term “Hybrid Manufacturing”. For instance, the query string used in Scopus was ‘TITLE-ABS-KEY (“hybrid manufacturing”)’. This helped maximize the number of retrieved articles. The articles were filtered based on the first instance, and then duplicates were removed, as shown in Figure 8. Some of the articles were excluded according to the following criteria: articles not in English; reviews or conference proceedings.

After careful examination, a total of 181 articles were included and compared in this review. Finally, the articles’ full texts were analyzed for their eligibility accordingly and were excluded if they lacked quality or had no experiments/testing performed (e.g., only focused on simulation).

## 3. Results from Literature Overview

**Figure 9:**
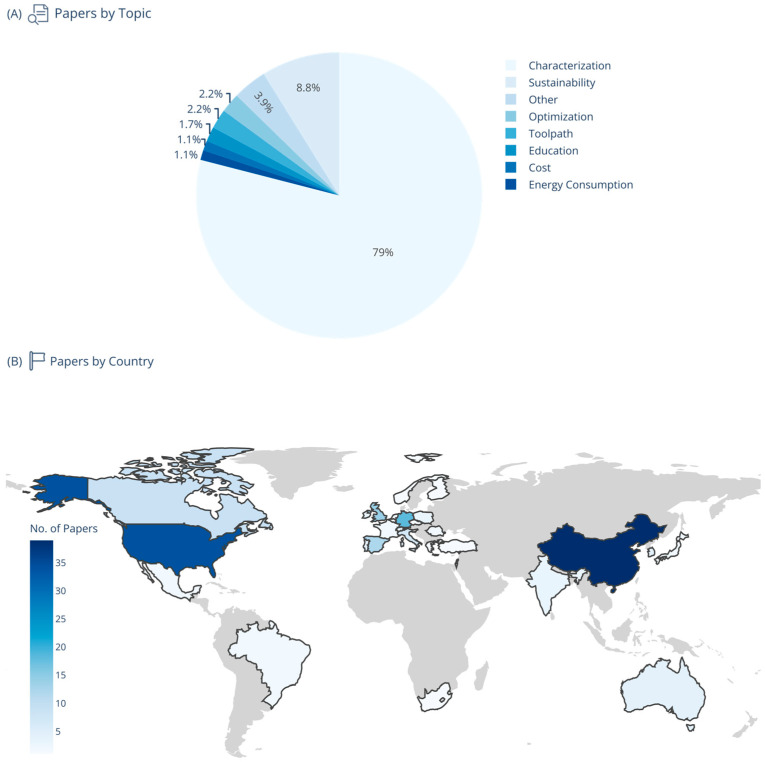
(**A**) Papers by research topic. The focus of most papers is on basic material characterization, including microstructure, chemical composition, thermal and mechanical properties, etc. (**B**) Papers by country.

**Figure 10:**
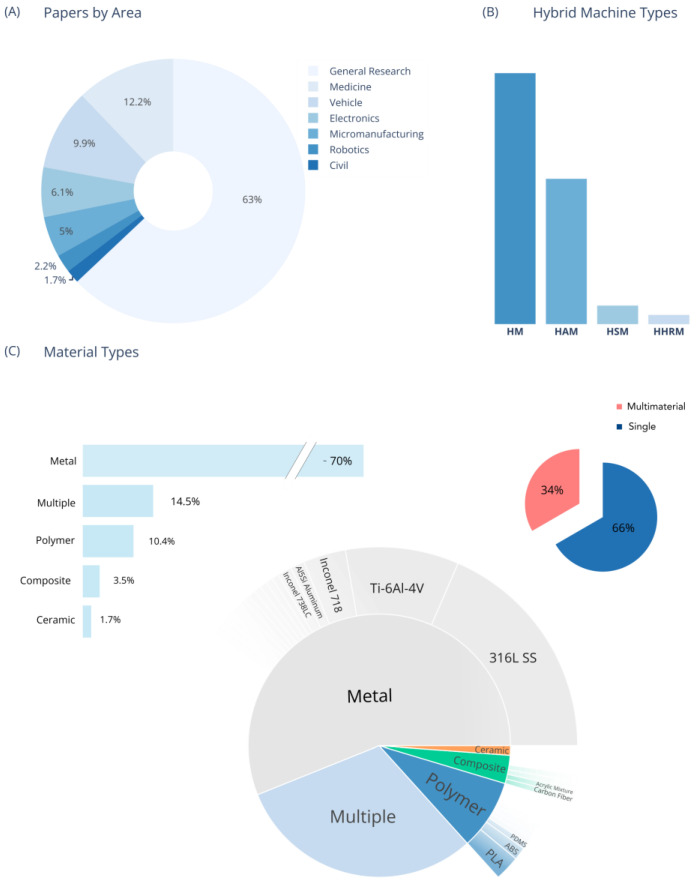
(**A**) Papers by area. (**B**) Hybrid machine categories. (**C**) Material types, including metal, polymer, composite (e.g., carbon fiber), ceramic, and multiple (combination of different material classes such as ceramic–metal, polymer–metal, etc.). The most common material type was metal (~70%), followed by multiple materials (14.5%), polymer (10.4%), composite (3.5%), and ceramic (1.7%). The sunburst chart presents the most common materials; for instance, the most used metal (more precisely metal alloy) is 316L stainless steel followed by Ti-6Al-4V. Single material parts are the most common (66%) compared to multi-material parts (34%). The label “multi-material” also includes metal-to-metal materials.

The results of the literature review are presented in Figure 9 and Figure 10, and information about each article is presented in Table 1. Extracted data included author, country, research area, topic, HM type, AM/SM processes, machine configuration, operation mode, material type, and multi-material characteristics. The characterization topics reflect the field’s priority to understand process operation and output, microstructures, and mechanical properties as foundational knowledge of hybrid integration technologies.

The assembled literature reveals several clear trends, which are briefly summarized in the following paragraphs. Geographically, the contributions span all major manufacturing hubs, with especially strong activity in China, the USA, Germany, and the UK; emerging work from Australia, Brazil, and Canada highlights growing global interest.

Across application domains, “Characterization” studies constitute the majority of investigations. In terms of areas of study, apart from “General Research”, notable fields include Medicine, Vehicles, Electronics, and Micromanufacturing, with smaller yet significant contributions in Robotics and Civil Infrastructure. Sustainability, optimization, and energy consumption each appear as dedicated topics in a handful of recent works, signaling a shift towards lifecycle and performance concerns beyond mere process feasibility.

Methodologically, nearly all systems employ a separate configuration (i.e., distinct additive and subtractive modules) and a sequential operation mode. Single-module or in situ hybridizations are less common, and only a few studies explore cyclical or concurrent approaches. On the additive side, Directed Energy Deposition (e.g., DED and LMD), Powder Bed Fusion (SLM/LPBF), and Wire-Arc AM (WAAM) dominate; subtractive processes most commonly involve milling and machining, with occasional etching, drilling, or laser-based finishing steps.

Material-wise, metals (particularly stainless steels like 316L, titanium alloys such as Ti-6Al-4V, and inconel grades) are the predominant focus, reflecting the biomedical, aerospace, and automotive drivers of hybrid manufacturing. Polymers and composites appear primarily in electronics and medicine contexts (e.g., FDM of PLA, SLA of resins), and only about one third of studies report true multi-material integrations. Ceramics and nanocomposites are explored sporadically, often in specialized micro-manufacturing or functional ink applications.

## 4. Hybrid Manufacturing Machines

The present discussion is focused on the types of hybrid machines or setups used in the reviewed studies. The materials and parameters used by some of these hybrid machines are discussed in the following sections.

### 4.1. Types of Hybrid Machines

**Figure 11:**
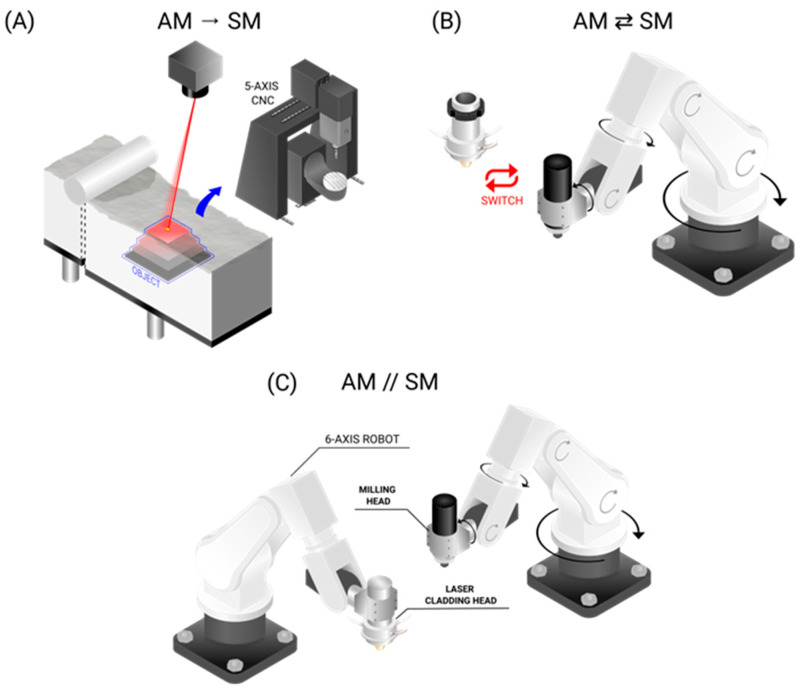
(**A**) Separate machines. After the part is produced (e.g., using SLS), it is then milled in a separate CNC machine. (**B**) Single hybrid machine. A robotic arm (or CNC machine) holds, e.g., the milling head, which can be switched to a laser cladding head. (**C**) Continuous hybrid machine. There are a deposition system and a milling system that work in parallel.

The collection of reviewed machine setups can be broadly classified into three types:

- **Separate machines**: This involves two separate machines, one for AM and another for SM, that are operated independently. This does not resemble a hybrid machine (Figure 11A).
- **Single hybrid machine**: This refers to a hybrid machine with changeable heads that work in shifts: one head for AM and another for SM. These can either be changed automatically or manually (Figure 11B).
- **Continuous hybrid machine**: This refers to a hybrid machine that can perform AM and SM synergistically. The AM and SM systems can work in shifts or simultaneously. The worktable can either be fixed or movable, functioning as an additional CNC machine for positioning of the part with respect to the laser head or milling tool. Instead of a milling tool, another laser can be used, and instead of a worktable, a powder bed may be used (Figure 11C).

**Figure 12:**
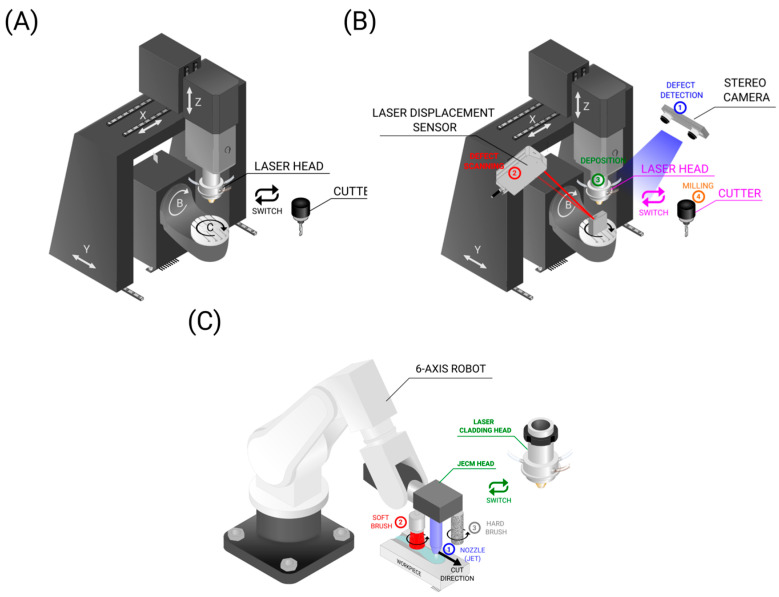
Single HM machines (with changeable AM and SM heads). (**A**) LMD/milling machine (laser head and cutter). (**B**) LMD/milling machine with sensors, 1—stereo camera, 2—laser displacement sensor, 3—laser head, 4—milling head (**C**) LMD/milling robot 1—the nozzle uses a jet for material removal, 2—soft brush to clean debris, 3—hard brush.

Elaborating on the different machine categories, the present section categorizes each unique machine found in the literature to provide a deeper understanding of the engineering behind them. Foreseeably, most of the analyzed setups consist of separate machines (Figure 11A) followed by single hybrid machines (Figure 11B) as the latter are relatively expensive compared to in-house solutions. Only a few studies use continuous hybrid machines, and CNC machines with three to five axes are in trend (Figure 11C) [25,28,37,40]. Figure 12 depicts some examples of separate machines. Some of these apparatuses fulfill AM, such as the customary SLM machine (Figure 12A) or WAAM (Figure 12B). One of these AM processes are then combined with one SM process, commonly CNC machining (Figure 11C) or the occasional use of WEDM.

SLM is a popular choice across the existing hybrid machine setups. SLM, SLS, or DMLS machines usually contain two axes found in the laser head (θ1 and θ2), corresponding to two mirror galvanometers (galvos) that enable the laser beam to scan the entire 2D plane of the powder bed. Additionally, the bed (worktable) has one axis (motor) that elevates the table every time a new layer is to be created (Figure 12A). For example, the M2 laser powder bed system uses a Yb-fiber laser [217,218]. One advantage of using a fiber laser such as Yb:YAG or Nd:YAG as opposed to a CO_2_ laser is the ability to better process aluminum, precious metals, and other highly reflective materials such as copper and brass. On the other hand, CO_2_ laser generators can produce higher powers at a lower cost. Thus, a CO_2_ laser is the preferred choice when cutting materials with an adequate absorbance such as steel [219]. Another competitive SLM machine, namely SLM 280HL, was utilized to create a workpiece with two sub-regions, a regular block combined with a lattice block, which was subsequently machined [220].

When using SLM, SLS, or DMLS, typically, a separate machine is needed to mill the part since sintering AM processes require special conditions (such as a powder bed) to build the part.

Usually, the part needs to be manually transferred from the AM machine to the SM machine, which is an issue in terms of operation time. This manual transfer introduces additional downtime due to transportation, fixturing, and realignment steps, which are prone to errors and may affect dimensional accuracy. In contrast, integrated hybrid machines perform automatic head/tool changes within a single setup, which, although requiring some time, is generally much faster and reduces handling risks. A fully integrated hybrid machine that can automatically change between AM and SM processes greatly reduces this production time and overhead to the manufacturer [26].

Another common setup observed throughout the studies is the single hybrid machine with switchable AM and SM heads, as presented in Figure 12A. In this setup, a laser cladding head (LMD) is frequently used as the AM head. The incorporation of sensors improves the automation and reliability of the machine (Figure 12B). A robot arm can be used instead to improve the workspace and provide flexibility and dexterity to handle complex components, as shown in Figure 12C [221].

Five-axis and six-axis LMD machines (robotic arm) as shown in Figure 12 are often capable of working with continuous axes. This contrasts with five-axis indexed machines (often regarded as three + two-axis machines), which need to start and stop between part regions. This means that using a five-axis continuous machine can improve the manufacturing time and may improve the quality of the part [222]. However, this type of machine will likely be more expensive since it uses more complex firmware and hardware. An example of this type of hybrid machine is the DMG LASERTEC65 3D (Bielefeld, Germany) [38,223,224]. The machine is composed of a five-axis machining center, a Siemens 840d NC controller (DMG manufacturer, Bielefeld, Germany), and a 2500 W fiber-coupled diode laser with a 3 mm laser spot diameter, coaxial deposition nozzle, and a metal powder feeder system.

Five-axis LMD machines offer the possibility of producing parts or modifying them, such as repairing a surface by depositing material or adding new features using AM. When producing parts from scratch, metal deposition via laser cladding (similarly to most 3D printing processes) is inherently slow compared to traditional processes such as die casting, and thus, it is often used for prototyping parts [3]. The additional time a Laser Cladding Deposition (LCD) system takes to produce a part from scratch translates to higher costs. However, the issue of traditional manufacturing technologies is the lack of tailoring of the parts for each specific case, since the diecast will produce the same part geometry in bulk. A relevant approach is to produce the part and most of its features using a traditional manufacturing process and then customize each part according to the user’s needs, be it a patient that requires a custom implant or an automotive industry client that requires a high-performance customized part. Therefore, five-axis LMD machines can be a viable option for the customization of parts, especially when combined with traditional processes.

Woo et al. used a machine with more axes (seven-axis machine) [32]. The kinematic redundancy of using seven axes (as opposed to five axes) may increase the workspace and improve the load (torque) distribution at the joints. However, since more actuators are used, the machine cost will naturally be higher [225].

**Figure 13:**
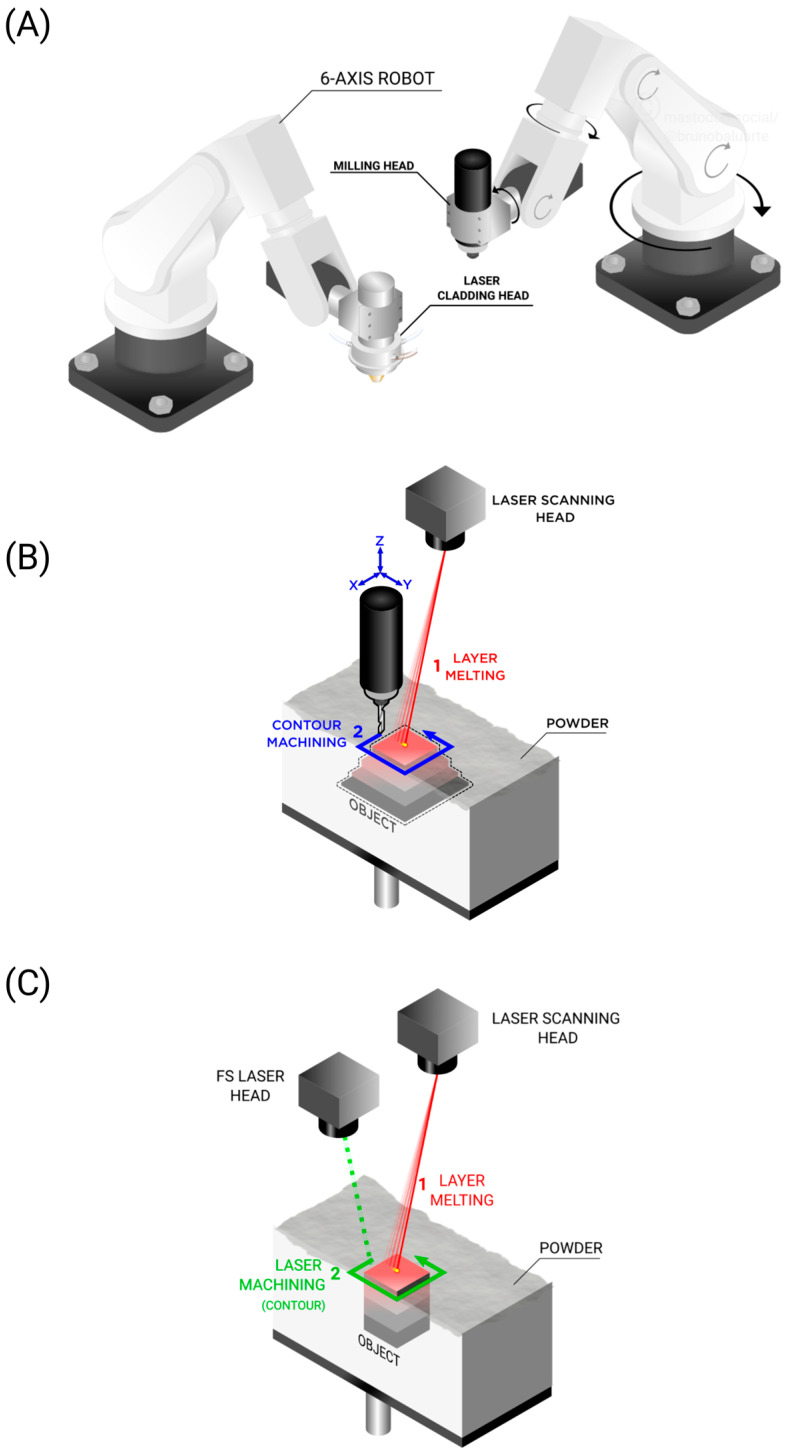
Continuous hybrid machines. (**A**) Two 6-axis robots fitted with milling and laser-cladding end-effectors. (**B**) SLM/milling hybrid cell showing layer melting followed by contour machining. (**C**) SLM/laser machining. Legend: red = layer melting/additive step; blue = contour or mechanical machining; green = laser contouring (femtosecond laser).

LMD samples, such as Inconel 718 components, can exhibit worse machinability. Analysis reveals that differences in machining arise from variations in material characteristics. LMD parts often have distinct microstructures and hardness levels, which affect chip morphology and increase cutting forces during milling [28]. Furthermore, the processing of the parts and addition of features will be slow, especially due to the fact that the machine axes (and laser head) have inherent inertia. To overcome this issue, an option is to create a faster system, namely a hybrid machine that works continuously. Examples of two robots operating in parallel are shown in Figure 13A, SLM and machine head working in turns are shown in in Figure 13B, and two laser heads working in turns are shown in Figure 13C.

**Figure 14:**
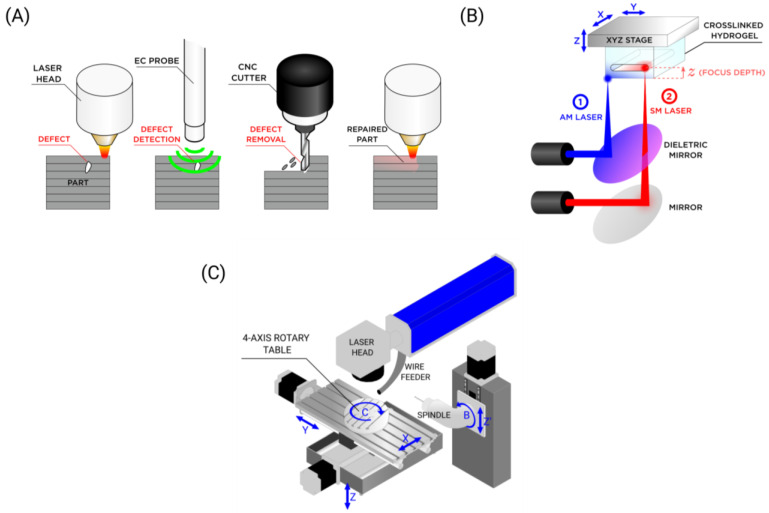
(**A**) LMD/machining with EC probe. Defect repair workflow: defect (red), defect detection sweep (probe), and defect removal with cutter and repair. (**B**) Hydrogel hybrid laser manufacturing. 1—additive step that crosslinks the hydrogel; 2—subtractive step using mirror and a dielectric mirror (**C**) Wire LMD (7-axis machine). This system combines a Hyundai WIA HiV560M (Changwon, Republic of Korea) five-axis vertical machining center with an independently controllable two-axis laser module. The laser module employed a Laserline high-power diode laser (LDM1000-100) (Mülheim-Kärlich, Germany).

Another option to improve the HM speed is to build a system that has a movable worktable (which secures the part) and a fixed laser head that scans the part surface, either to add or remove material. Since the laser head is composed of two galvo mirrors that have low inertia, the machine can quickly move the laser to target distant areas of the part. After scanning one region, the movable worktable can reposition the part to scan another region. This approach can be particularly useful for small to medium components since their low inertia allows the worktable machine to move faster compared to a five-axis machine that includes a laser or machining head that needs to be carried. Other examples of hybrid machines reviewed in this study are presented in Figure 14.

Most studies reviewed in this paper present hybrid technologies that deposit material in a layer-by-layer fashion, where each layer is planar. However, some machines from the studies, particularly the DED/LMD machines, have the potential to deposit material in complex curvilinear surfaces [223]. With respect to materials, most of the additive laser manufacturing machines presented in the studies only use metallic materials. This may be restrictive for applications in fields such as biomedical and aerospace, where multi-material parts are relevant.

The hybrid technology setups across studies typically do not constitute an integrated machine that is capable of handling both additive and subtractive manufacturing seamlessly. The setup requires the user to either manually transfer the part from one machine to another or manually switch heads of the hybrid machine. Studies that use a single hybrid machine or setup to handle both processes are included [4,28,209,223,226,227]. Most of these are based on exchanging heads automatically, and others have two distinct additive and subtractive subsystems that work in shifts. Ideally, the hybrid machine would perform AM and SM processes synergistically or continuously; however, for most studies, this is not a necessity.

Hybrid machines may only use a laser for the manufacturing process. Generally, one laser head handles the additive process, and the other handles the subtractive process [32,35,36,37,40]. As already discussed, this has the potential advantage of processing parts faster due to the reduced inertia of the machine (there is less machinery to move). However, one disadvantage is that the laser may not be suitable for larger cuts unless a high-power laser, in the kilowatts (kW) order, is used in the SM process.

Just a few setups from the analyzed studies are capable of modifying an already existing component [190,213,223]. Here, modification involves performing additive or subtractive operations over the existing surface of the part. For instance, it is possible to repair an already fabricated part and deposit stainless steel along a curved surface. However, only one type of material was utilized [223]. The deposition and removal of material from a complex surface shows the potential of hybrid technology to customize existing parts according to the user’s needs. The market is increasingly demanding the fabrication of custom components, for instance, the production of patient-specific implants, custom sports equipment, or optimized parts with high resistance to weight ratio for aerospace and automotive applications [228]. A competitive advantage in the field can be obtained by modifying standard parts using hybrid technology and combining two or more materials. Thus, HM combined with ingenuity can help optimize the parts’ mechanical performance, stability, compatibility (e.g., osseointegration), durability, and other characteristics [37]. HM also provides the possibility to include electronic elements inside the parts, such as sensors and piezoelectric material [229]. By increasing the tailoring of the part, the designer can achieve specific project needs and better integration into other systems, thus increasing the added value to the customer.

Manufacturing of implants and biomaterials is a field of extreme relevancy. HM opens the door to the creation of organic surfaces, cellular microstructures, and bioactive materials. Medical-grade titanium alloy (Ti6Al4V) specimens can be manufactured using SLM and further modified using femtosecond laser ablation as a subtractive step. This hybrid approach offers the opportunity to produce titanium endosseous implants with optimized surfaces for enhancing osteogenic differentiation of human mesenchymal stem cells [37]. Furthermore, the studies modified the surface of zirconia implants to include the biomaterial hydroxyapatite (Hap) using Nd:YAG laser and CO_2_ laser for SM and AM, respectively [35,36]. As observed across multiple studies, it is important to keep an inert atmosphere when processing metallic parts (such as titanium). This is especially true when dealing with biomedical applications and PBF processes. Laser cladding heads (DED process) also deposit metal using a shielding gas, usually argon [42]. Still, working with a laser head (SLM) and a milling head in shifts seems to be the customary approach instead of having two laser heads operating alternately [28,230].

### 4.2. Hybrid Machine Modes of Operation

The review classifies HM processes as either concurrent or sequential. Herein, we only consider “concurrent” processes as those that truly have AM and SM processes working at the same time [231]. Henceforth, if they work almost at the same time, but in shifts, they should be considered “cyclical”. For instance, the AM process creates one layer, then the SM process finishes that layer, and then this sequence repeats cyclically until all the layers of the part are finished. If there is only a sequence of steps that are not cyclical, then the process should simply be called *sequential*.

*Concurrent (Concurrent Mixed)*: The two processes manufacture the part concurrently, such as through two robot arms, with one using AM and the other using SM. They can work in parallel.

*Assisted or Coupled*: This process is also concurrent, but the secondary process only assists the primary process, and it is not a full process. This is a typically “coupled process”, such as a laser coupled with milling in a single head that deposits and mills along a direction.

*Sequential*: The two machines work in separate processes without integration. The part is fabricated using, e.g., an AM machine and then is transferred to the SM machine. This process can be performed manually or automatically.

*Manual*: At the end of the first manufacturing process (e.g., AM), the operator manually transfers the sample or object to the other machine (e.g., SM).

*Cyclical*: Like the “sequential” process, a layer of the part is created using AM, and then SM is applied to that layer, and this sequence repeats for each layer; hence, it is considered cyclical. It is also like the “concurrent” process in the sense that the two processes operate almost at the same time and in situ, but they are not truly concurrent. We essentially have two machines (or two heads) that work in shifts.

## 5. Hybrid Manufacturing Categories

At present, there are several HM technologies which can be roughly grouped into four main categories. Typically, the traditional definition of HM or hybrid additive–subtractive manufacturing (HASM) is the combination of additive and subtractive processes, and this is one category. However, in the literature, several authors refer to any combination of manufacturing processes that are integrated to obtain a final product as a “hybrid manufacturing process”. Hence, when studies hybridize two AM processes, this is proclaimed as Hybrid Additive Manufacturing (HAM). If the two processes are subtractive in nature (SM), this is referred to as Hybrid Subtractive Manufacturing (HSM). Another modern category was coined herein as Hybrid Human–Robot Manufacturing (HHRM), where the operator manufactures or assembles a part in parallel with a robot. This is an important step in the push for Industry 5.0, where humans shall have a crucial role in the integration of the processes that add value to the business.

As seen in Figure 10B, traditional HM is still the most common category across studies, followed by HAM, with only a few studies dealing with HSM and HHRM. This presents an opportunity to develop specific hybrid systems based on HM involving both robot and human cooperation, where the operator is guided via AR goggles or projections to maintain, oversee, or fix systems that are automated via Artificial Intelligence (AI). The human element, expanded by AR and AI, plays a crucial role in this manufacturing chain equivalent to a high-dexterity robot with many Degrees of Freedom (DOFs).

Another important concept that deserves a notable mention is the manufacturing of multi-material parts. This might be referred to as multi-material additive manufacturing (MMAM). Hybrid technologies based on this type of manufacturing can truly open the door to the modern production and chemical customization of high-end parts that meet ever-increasing client requirements.

## 6. Manufacturing Processes

In the HM literature, DED is the dominant AM process, followed by PBF technologies (such as SLM). This naturally correlates with the fact that most studies used metal as their raw material. SLM is tailored to only manufacture parts from scratch, which can be a considerable shortfall if the intention is to customize existing parts. Not coincidentally, a myriad of hybrid machines are based on DED, especially for part repair or customization. The advantage of DED is that not only can it manufacture a part from a build platform, but it is also capable of adding material on top of existing surfaces or parts. Therefore, DED bears some crucial advantages, such as part coatings, repair, and modification. Other advantages include the production of larger parts and greater mechanical properties compared to SLM. Notwithstanding, the DED machine firmware needs to be diligently programmed using the inverse kinematics of the machine and the distance between the tool and the part. Moreover, DED machines provide a wide range of feedstock material. The machine’s feeder, which contains the material, can be replaced or combined to produce multi-material parts (e.g., metal–ceramic composites) [232].

In contrast, the advantage of using SLM over DED processes is the higher control of the layer thickness since the powder is uniformly spread by the machine and the process is always performed on a flat region. Despite DED allowing for manufacturing in curved regions, it still lacks precise control of the layer’s thickness even if performed on a flat surface because of the variable bead height from the melt pool. This limitation can be somewhat overcome using a milling machine, which is a process beneficial to SLM, to improve the surface quality. SLM also leverages higher dimensional resolution and powder recyclability. Furthermore, shrinkage and residual stresses are possible issues while using DED [232]. Additionally, surface finish in DED is traditionally lower compared to processes like SLS. As an example, using 316L as the basis for comparison, the SLS study achieved a surface roughness (Ra) lower than 10 μm [233]. On the other hand, the laser cladding (DED) study produced a surface roughness, Ra, of ~15 μm [234]. Either way, both SLM and DED usually require surface finishing to produce high-fidelity parts [6]. Furthermore, DED systems (especially hybrid DED) are usually more expensive than SLM systems. One disadvantage of DED is the lack or complexity of support structures that traditional FDM systems offer. SLM intelligently uses unmelted powder as the support for the next layer. Dissolvable support structures or complex slicing algorithms for overhangs are solutions for the DED support structure crisis [235,236].

Most of the analyzed studies used conventional machining (such as milling or turning), whereas just a few used high-speed milling (HSM) or laser machining [27,29,32,34,35,36,37,39,40,102,159,237,238,239,240,241,242]. Additionally, it should be emphasized that alternative methods were used for machining, such as micro milling, cryogenic milling, and JECM [25,41,72].

Compared to traditional machining, HSM allows for material to be removed at higher rates, meaning it improves manufacturing/finishing efficiency and thus lowers the cost. Furthermore, since the spindle’s tool is working at a higher speed, the generated forces are lower, and less heat is generated. This is useful to avoid distortion of the part and deflection of the tool. However, by working with the tool at higher speeds, more precisely at higher acceleration/deceleration rates, an HSM machine will have higher wear of guideways and spindle bearings, and therefore, substitution of the milling tool is often required. Furthermore, HSM machines require a specialized spindle, fixtures, controllers, and materials to handle delicate tasks, which lead to higher maintenance costs [243].

EDM is a non-contact type process where the wire (or electrode in the case of μ-EDM) does not touch the workpiece. Therefore, the machinist is able to generate slots, grooves, and other features without applying stress to the part. Furthermore, in contrast to conventionally drilled surfaces, an EDM surface will be smooth without burrs. Some limitations of EDM include the inability to machine non-conductive materials, 3D curved surfaces, impenetrable surfaces, and a low metal removal rate [240].

Furthermore, the benefit of using laser machining compared to traditional machining is its speed and precision with which one can scan a surface. It allows for the automation and application of patterns onto the surface. Some studies used ultrashort pulse laser micromachining with a duration in the femtosecond scale [37,40]. This technique has the advantage of using cold and contactless processing [244]. The disadvantage is that laser processing is usually focused on surface modification, whereas conventional milling allows for cutting larger portions of material.

**Figure 15:**
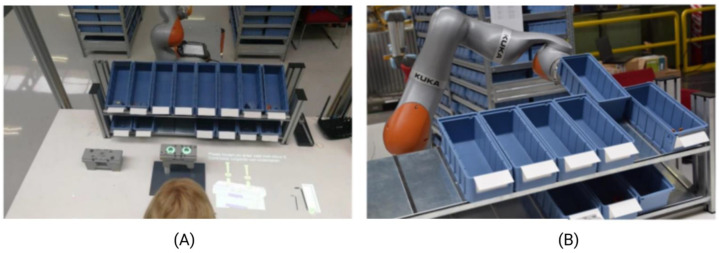
Hybrid assembly at a manufacturer specialized in producing telescopic slides. The goal is to orchestrate a smart factory across all production areas to reduce the dependency on the operator’s experience during the tool preparation phase. (**A**) Augmented reality (AR) is used to help even the non-experienced operator during the laborious assembly of specialized tools. (**B**) The companion mobile robot chooses and places necessary parts on the workstation while the operator assembles the part (photographs adapted from Ref. [189]).

In the age of AI breakthroughs, especially with developments accelerated by OpenAI, the manufacturing paradigm will inevitably mutate into a situation where intelligent machines will cooperate with humans to develop bleeding edge parts efficiently and safely. This shall establish the eventual shift towards Industry 5.0 that builds upon the IoT processes of Industry 4.0 combined with AI to maximize the cooperation between humans and robots, as shown in Figure 15. The following collection of articles present HHRM-specific research contributions to the field and focus on Industry 4.0, IoT, and Cloud, emphasizing significant advancements in manufacturing efficiency and capability [83,166]. The integration of human expertise with flexible robots also aligns with Industry 4.0 trends, focusing on smart manufacturing solutions [189,211]. This suggests a focus on improving robot learning capabilities through human interaction, potentially leading to more intuitive and efficient human–robot collaborations.

Comparing these HHRM-based studies with other machine types like HM and HAM reveals some key differences. In HM, HAM, and HSM processes, more emphasis is placed on technological integration for material processing rather than on the human–robot collaboration aspect seen in HHRM studies. HHRM systems, with their emphasis on human–robot interaction, fill a distinctive gap in the field of HM. These can be particularly helpful in an assembly context and may also be important for outsourcing heavy loads from the user to the robot, improving the operators’ well-being and reducing health risks. Symbiotically combining all of these systems will pave the way for smart warehouses that will become highly efficient and provide end customers with services or products at lightning speed for a fraction of the typical cost via economies of scale.

## 7. Raw Materials and Manufactured Parts

As can be analyzed using the graphs in Figure 10, most studies use metal as their base material, followed by using multiple material combinations such as metal–metal, metal–polymer, etc. Several studies combined two or more materials, effectively producing a multi-material part [41,78,92,190,241,245]. However, most of these studies present the combination of a powder material with similar characteristics compared to the substrate material (such as the combination of two alloys). Only a few studies combined materials with considerably different chemical compositions [33,35,36,229,237]. Silver paste may be combined with a PTFE substrate, with a focus on electronic systems [237]. Injection molding inserts were produced by aggregating steel with a copper alloy. C5 (XC48) steel, Ampcoloy 83 copper alloy, and X3NiCoMoTi maraging steel were combined to produce these parts [33]. Carvalho and Faria et al. combined zirconia and hap with the intent to biofunctionalized dental implants for better osseointegration [35,36]. Finally, 13-8 PH stainless steel, ceramic ink, and silver and platinum particles were amalgamated to produce smart parts that contained electronic components between layers [229]. Some studies utilized polymeric materials like PLA (Polylactic Acid) deposited with a Fused Deposition Modeling (FDM) head, PEGDA was cross-linked using a femtosecond laser (FS laser) modulated using a digital micromirror device (DMD) to cross-link only the desired regions, and a PTFE fluoropolymer substrate was dispensed by silver paste with a 3D dispenser printer [40,227,237].

**Figure 16:**
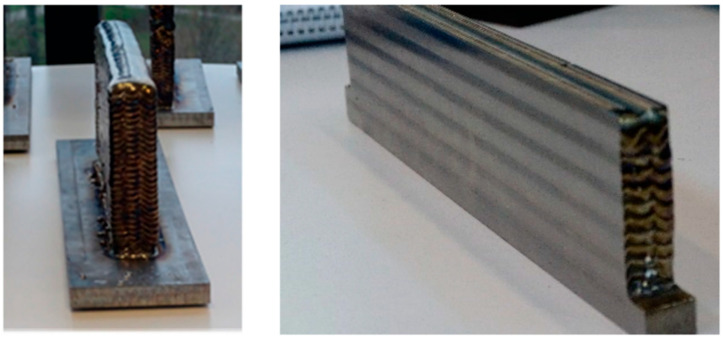
TI6Al4V walls produced via Wire Arc Additive Manufacturing (WAAM), specifically Plasma Arc Welding (PAW). Macrographs and microstructures of the wall manufactured by PAW-WAAM are presented in the study for the different directions (photographs adapted from Ref. [194]).

A large number of studies utilized 316L stainless steel, which is a medical and marine-grade material [27,38,92,159,223,246]. Moreover, Ti-6Al-4V titanium alloy is also popular and is often deployed in the aerospace and biomedical industries owing to its low density and high corrosion resistance [29,217]. Li et al. used PLA as the base material, which was deposited via an FDM head that can be switched with a laser cladding head [227]. An example of a study that used a single material for the HM process, namely Ti-6Al-4V, is shown in Figure 16.

Only a handful of studies performed the deposition of material along curved surfaces [62,223,227]. The remaining studies built the components either from a powder bed (in the case of SLS and SLM processes) or from scratch using a simple plate as the base, also referred as substrate. Some of the laser cladding studies showed that the inclination of the base plate negatively influences the deposition of the material Therefore, it is convenient to program the system in such a way that the DED head deposition axis is as perpendicular as possible to the part’s surface. This way, the melt pool does not slip as much to the side and maintains its integrity [102]. Nevertheless, some DED systems have the part fixed to the base table, i.e., the DED head may deposit the material at non-ideal angles in some regions. By having a movable table that can position the part at proper angles with respect to the DED head, the designer can produce parts whose geometry is closest to the original CAD model. Having two systems, namely one that moves the part and the other that moves the laser head, also has the benefit of allowing the head to deposit material in opaque regions of the part (e.g., the bottom region of the part). By moving or tilting the part, it is possible to expose those regions and thus customize the part.

In terms of material properties, 316L parts can be manufactured using ASHM with different laser energy densities ranging from 159 J/mm^3^ to 370 J/mm^3^. With lower laser energy densities, the specimens obtained a Yield Strength (YS) of ~380 MPa and an Ultimate Tensile Strength (UTS) of ~563 MPa [92]. With higher laser energy densities, the YS was ~405 MPa and the UTS was ~570.5 MPa. In terms of hardness (Vickers microhardness, HV), lower laser energy densities exhibited a value of 201 HV, whereas higher laser energy densities achieved 212 HV. Generally, higher energy input results in increased density and reduced porosity. HM can produce materials containing hard surfaces. The hardness of the ASHMed top and side surfaces is 12.5% to 14.1% higher than that of the SLMed samples [78]. The cutting forces experiment between the wrought samples and SLMed samples showed that SLM can present a higher cutting force up to ~30% due to a finer microstructure, which relates to a higher yielding strength.

It is possible to create stronger parts using powder and LMD compared to traditional ingot parts. A study using 316L-Si observed that compared to the 316L-Si ingot, the LMDed sample showed higher strength due to small powder grains [241]. The ingot sample had a YS of ~170 MPa and a tensile strength of ~485 MPa compared to YS values of 451 MPa and 693 MPa. In some cases, machining a part and then adding features on top may decrease the part’s overall strength. The study showed that the mean flexural strength of ASHMed samples of Zirconia + HAp laser textured samples was 503 ± 24 MPa, lower than those reported in the literature for the range of (692 ± 41 MPa) without the AMed part (HAp) [36].

**Figure 17:**
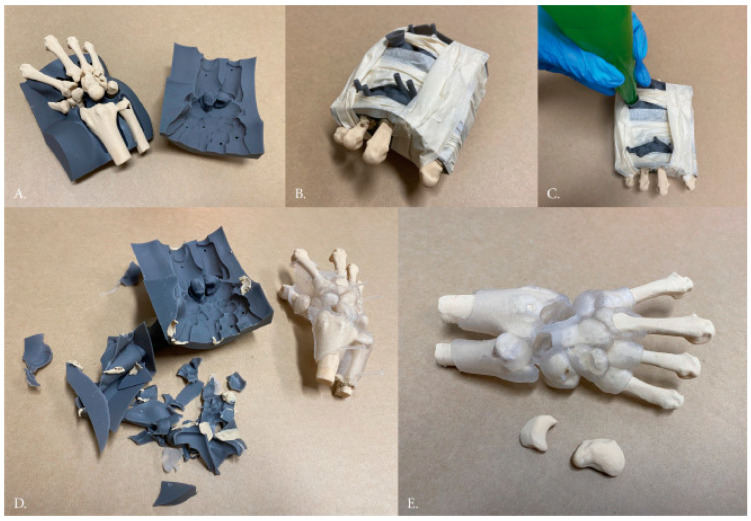
Development, via 3D printing, of a novel surgical practice rig to address the lack of simulation models for scapholunate interosseous ligament (SLIL) injuries. (**A**) Bones are positioned within a 3D-printed resin mold such that there is space to allow for the pouring of silicone around them. (**B**) The resin mold is securely sealed and wrapped in tape to prevent excessive silicone overflow during casting. (**C**) Silicone is manually injected into the mold using a piping bag, akin to an injection molding process. (**D**) Following silicone curing, the mold is broken to retrieve the final model. (**E**) Any excess material from the molding process is manually removed from the final model (photographs adapted from Ref. [87]).

The bonding strength of printed patterns to substrates can be higher than the filler material itself. For instance, EDM was used to produce 17-4PH surfaces with a roughness of 2.5, 1.4, 0.8, and 0.4 μm, onto which 316L was printed on top and a bonding strength of ~600 MPa was observed [27]. There are also studies that use hybrid manufacturing and multi-material approaches for education purposes, as shown in Figure 17, based on resin and medical-grade silicone GSM50 [87]. Sometimes, the mechanical strength of the parts is not a major concern; rather, their texture and color are significant factors such that they replicate the biological systems or structures of interest [183,200]. The toolpath is a crucial factor for conventional and modern age manufacturing processes. Hence, the work intends to cover different aspects of the toolpath.

## 8. Recent Advances in Hybrid Manufacturing

Toolpath generation for hybrid additive–subtractive manufacturing builds on the evolution of traditional CAM strategies, where simple geometric approaches gave way to advanced automation and data-driven methods. Early work established that layer slicing, iso-parametric, and iso-scallop methods could balance surface finish against cycle time and tool wear [247]. Modern refinements leverage the pre-processing of point clouds to reduce redundant motion and machine learning models trained on past trials to predict optimal feed, speed, and stepover settings [248,249]. These techniques form the backbone of advanced hybrid systems by ensuring that deposition and removal operations can be planned coherently, minimizing both residual errors and unnecessary machining passes.

Pushing beyond three-axis operations, accessibility-driven and visibility-driven planning algorithms assess tool orientations to avoid gouging and maintain ideal contact angles, a capability first honed in five-axis machining [250]. In hybrid contexts, Thien et al. introduced on-machine probing for wire-based directed energy deposition tools, sampling the as-built surface via touch probes and fitting triangular, trapezoidal, or hybrid geometric models to guide subsequent milling. This closed-loop strategy yielded up to a 68% reduction in machining time and significant improvements in surface roughness by explicitly correcting deposition irregularities during the subtractive stage [184]. Hamilton et al. expanded the approach with a large-scale robotic testbed—combining pellet extrusion, wire–laser DED, and milling under a unified ROS framework—to tune toolpath parameters in real time based on force and geometry feedback, demonstrating the feasibility of sensor-driven hybrid workflows for complex, large-format parts [251].

Building on these frameworks, Chen et al. proposed a scalable, concurrent trajectory optimization scheme for robot-assisted AM, which refines an input toolpath by segmenting it into smaller blocks and jointly optimizing tool orientation, kinematic redundancy, and waypoint timing via parallel Sequential Quadratic Programming (SQP) [252]. Despite these advances, hybrid toolpath planning remains computationally intensive: high-resolution voxel and point-cloud models impose heavy GPU and CPU loads in real time [248], and achieving optimal trade-offs among machining time, surface integrity, and form accuracy continues to challenge multi-objective optimization frameworks [247]. Future work will need to integrate robust automation with data-driven optimization to fully exploit the flexibility of hybrid additive–subtractive platforms.

Complementing these trajectory optimization and hybrid planning strategies, recent work has begun to embed multidisciplinary processes into AM frameworks. Török and Dupláková (2025) propose an integrated practical framework for multidisciplinary prototype design and manufacturing processes, which systematically integrates ergonomic evaluation, reverse-engineering methodologies, virtual comfort testing, and Multi Jet Fusion (MJF) to yield personalized prototypes with demonstrably improved comfort (e.g., “comfort” index = 30.3) while slashing development time and cost through early-stage digital validation [253]. To tackle material waste in FDM workflows, Török et al. (2024) designed and prototyped a pair of specialized welding pliers that seamlessly join thermoplastic filaments used in FDM printers; they achieved this by mapping optimal welding temperatures for various polymer grades and verifying weld tensile strength [254]. Together, these studies illustrate the possibility of embedding ergonomic, materials science, and tool design expertise into HM pipelines. This type of technology will drive sustainable, user-centered innovations, paving the way for future hybrid platforms that integrate path planning with on-the-fly tool adaptation and feedback-guided process control.

Recent studies have further expanded the frontiers of HM by integrating AI, human–robot collaboration, and smart digital infrastructures. As Industry 5.0 principles, emphasizing personalization, resilience, and human centricity, gain traction, HM is increasingly seen as a cornerstone of next-generation production strategies. These developments not only enhance efficiency but also open pathways toward more sustainable, adaptive, and flexible manufacturing systems tailored for complex, high-value components.

The use of real-time, AI-driven closed-loop control is a recent development in this field. Baswaraju Swathi et al. implemented a hybrid deep Convolutional Neural Network (CNN) architecture integrated with an LPBF system to autonomously detect and correct process anomalies. Their framework achieved an F-score of 93.8% in defect detection and control–actuation frequencies exceeding 10 Hz, demonstrating significant improvements in surface finish and in-process correction within a single build [255].

Within the Industry 5.0 paradigm, Anang et al. explored AI-enhanced human–machine collaboration. Their work showed how predictive analytics and real-time data processing can augment operator decision-making, with case studies across the automotive and electronics sectors reporting measurable productivity gains [256]. Jing Xu et al. further advanced this vision by proposing a human-centered framework based on Cyber–Physical–Social Systems (CPSSs). Their survey outlined the convergence of embodied intelligence, large language models, and cloud computing under the Industry 5.0 umbrella with collaborative swarms of robots, sensors, and LLM agents enabling distributed learning and autonomy at scale [257]. Su et al. introduced a three-layer knowledge graph architecture for digital twins in aero-engine blade manufacturing. By integrating physics-based models with real-time sensor data, researchers achieved a prediction precision of 77.4%, surpassing conventional and deep-learning-only approaches. This highlights the effectiveness of hybrid digital–physical integration for predictive quality assurance [258]. An intelligent process-planning system for five-axis hybrid directed energy deposition (DED) and milling machines was proposed. Their curve-based partitioning method streamlined planning and execution, underscoring the role of intelligent planning in advancing HM productivity [259]. These developments point toward several emerging directions:

Personalized Manufacturing: This enables on-the-fly customization of individual parts through AI feedback.

Collaborative Manufacturing Networks: These involve the use of hybrid machines as intelligent nodes in decentralized production ecosystems.

Multi-Material and Functional Grading: This involves embedding tailored mechanical, electrical, or thermal properties within single components.

Self-Optimizing Systems: These involve leveraging reinforcement learning and swarm intelligence for real-time process optimization without human intervention.

## 9. Conclusions

Hybrid manufacturing is rapidly evolving into a transformative support of advanced manufacturing, combining the precision of subtractive methods with the flexibility of additive techniques. However, despite remarkable progress, current hybrid systems often lack essential capabilities such as real-time adaptive control fully integrated with artificial intelligence. For accurate adaptiveness, machines must harness rich sensor inputs, such as stereo vision cameras and displacement and temperature sensors, processing them automatically to guide and self-correct operations toward optimal outcomes.

Moreover, many existing systems remain only partially integrated into Industry 4.0 and 5.0 frameworks that stray from the full potential of Internet of Things connectivity, data automation, cloud computing, and intelligent decision-making. The integration of these technologies represents a significant opportunity for future development. Looking ahead, hybrid manufacturing will increasingly embrace smart materials, 4D printing concepts, and seamless collaboration between humans and machines. With the aid of artificial intelligence and augmented reality, the role of the human operator will shift from executing tasks to supervising, innovating, and creatively engaging with production systems. This association promises to liberate workers from repetitive labor to unlocking unprecedented levels of customization, functionality, and efficiency in component design and fabrication.

## Figures and Tables

**Table 1:** Systematic review.

Multi-Material	Material Type	Material	Other Process	SM Process	AM Process	Operation	Configuration	Type	Topic	Area	Country	Citation Key
Yes	Composite	Carbon Fiber, Epoxy	Hand Layup	-	Automated Fibre Placement (AFP)	Sequential	Separate	HAM	Characterization	Vehicle	Australia	(Air et al., 2023) [43]
No	Metal	Inconel 718	Wrought	-	Directed Energy Deposition (DED)	Sequential	Separate	HAM	Characterization	Research	UK	(Al-Lami et al., 2023) [44]
No	Metal	Stainless Steel (SS)	Single Point Incremental Forming	-	Selective Laser Sintering (SLS)	Sequential	Separate	HAM	Characterization	Research	Italy	(Ambrogio et al., 2019) [45]
Yes	Polymer, Composite	GPET, LCP Fiber	Compression Molding	-	Fused Deposition Modeling (FDM)	Sequential	Separate	HAM	Characterization	Research	Poland	(Andrzejewski et al., 2022) [46]
Yes	Metal	AA 2219, AISI 321	Friction Surfacing	-	Cold Metal Transfer (CMT) Welding	Sequential	Separate	HAM	Characterization	Research	India	(Babu et al., 2019) [47]
No	Metal	6511 SS	Precision Machining (PM)		Selective Laser Melting (SLM)	Sequential	Separate	HM	Characterization	Research	China	(Bai et al., 2020) [48]
No	Metal	Ti-6Al-4V	Metal Forming	-	Wire Arc Additive Manufacturing (WAAM)	Sequential	Separate	HAM	Characterization	Vehicle	Germany	(Bambach et al., 2020) [49]
No	Metal	AA 6016	Hole-Flanging Forming	-	Directed Energy Deposition (DED)	Sequential	Separate	HAM	Characterization	Vehicle	Switzerland	(Bambach et al., 2021) [50]
No	Polymer	VisiJet® M2 RWT		Machining	MultiJet Printing (MJP)	Manual	Separate	HM	Characterization	Medicine	USA	(Basinger et al., 2018) [51]
Yes	Ceramic	Porcelain, Graphite Ink	Direct Ink Writing (DIW)	Machining	Layerwise Slurry Deposition	Sequential	Single	HAM	Characterization	Electronics	Germany	(Bernardino et al., 2020) [52]
Yes	Metal, Composite	Structural Glass, Silver Ink		Laser Ablation (LA)	Inkjet Printing	Sequential	Separate	HM	Characterization	Electronics	Australia	(Beziuk et al., 2019) [53]
No	Metal	AA6082		Machining	Hybrid Additive Manufacturing (HYB-AM)	Sequential	Separate	HM	Characterization	Research	Norway	(Blindheim et al., 2019) [54]
Yes	Metal	AlSi10Mg, Al99.8	Forging	-	Laser Powder Bed Fusion (LPBF)	Manual	Separate	HAM	Characterization	Research	Germany	(Bohm et al., 2020) [55]
No	Metal	316L SS		Machining	Directed Energy Deposition (DED)	Sequential	Separate	HM	Characterization	Research	Brazil	(Bordinassi et al., 2022) [56]
No	Metal	316L SS	Polishing, Grinding	Machining	Directed Energy Deposition (DED)	Sequential	Single	HM	Characterization	Research	USA	(Botcha et al., 2020) [57]
No	Metal	Ti-6Al-4V	Femtosecond Laser Ablation (FSLA)		Additive Manufacturing (Laser-Based) (AM-LB)	Cyclical	Separate	HM	Characterization	Medicine	France	(Bouet et al., 2019) [37]
No	Metal	Aluminum (Al)		Machining	Ultrasonic Additive Manufacturing (UAM)	Cyclical	Separate	HM	Characterization	Electronics	UK	(Bournias-Varotsis et al., 2018) [58]
No	Metal	Inconel 718		Machining	Selective Laser Melting (SLM)	Sequential	Separate	HM	Characterization	Research	USA	(Brown et al., 2018) [59]
No	Metal	316L SS	Laser Re-melting	-	Directed Energy Deposition (DED)	Sequential	Separate	HAM	Characterization	Research	Italy	(Bruzzo et al., 2021) [60]
Yes	Polymer, Composite	Glass Fiber, PLA	vacuum forming	Machining	Fused Deposition Modeling (FDM)	Sequential	Separate	HM	Characterization	Research	UK	(Butt et al., 2019) [61]
No	Metal	Inconel 718		Machining	Laser Cladding Deposition	Sequential	Separate	HM	Characterization	Research	Spain	(Calleja et al., 2018) [62]
No	Metal	AISI H13		Machining	Wire Arc Additive Manufacturing (WAAM)	Sequential	Separate	HM	Sustainability	Research	Italy	(Campatelli et al., 2021) [63]
Yes	Metal	Eutroloy 16606A, 42CrMoS4		Machining	Laser Metal Deposition (LMD)	Sequential	Separate	HM	Sustainability	Research	Spain	(Castro et al., 2018) [64]
-	-	-		Machining	Electron Beam Melting (EBM)	Sequential	Separate	HM	Toolpath	Research	USA	(Chen et al., 2018) [65]
No	Metal	316L SS	Welding	-	Laser Powder Bed Fusion (LPBF)	Sequential	Separate	HM	Other	Civil	Italy	(Chierici et al., 2023) [66]
Yes	Metal, Polymer	PC, ABS, PLA, TPLA, Silver Paste	Material Extrusion (ME)	-	Direct Writing (DW)	Sequential	Separate	HAM	Characterization	Electronics	UK	(Cicek et al., 2023) [67]
-	-	-		Machining	Wire Arc Additive Manufacturing (WAAM)	Sequential	Separate	HM	Characterization	Research	USA	(Cornelius et al., 2022) [68]
Yes	Metal	Inconel 718, MetcoClad 718		Machining	Directed Energy Deposition (DED)	Sequential	Separate	HM	Characterization	Research	Spain	(Cortina et al., 2018) [69]
No	Metal	316L SS	single-point incremental forming, (SPIF)	-	Wire Arc Additive Manufacturing (WAAM)	Sequential	Separate	HAM	Characterization	Research	Portugal	(Cristino et al., 2021) [70]
No	Metal	Ti-6Al-4V	Forging	-	Laser Metal Deposition (LMD)	Sequential	Separate	HAM	Characterization	Research	China	(Cui et al., 2021) [71]
No	Metal	316L SS	Micro-End Milling (MEM)		Powder Bed Fusion (PBF)	Sequential	Separate	HM	Characterization	Research	Brazil	(de Assis et al., 2020) [72]
Yes	Composite	Al 1050, β-SiC	Cryorolling	-	Stir Casting	Sequential	Separate	HAM	Characterization	Research	India	(Deb et al., 2018) [73]
No	Polymer	Polydimethylsiloxane (PDMS)	Two-photon polymerization (TPP or 2PP)	-	Stereolithography (SLA)	Sequential	Separate	HAM	Characterization	Micromanufacturing	Belgium	(Dehaeck et al., 2019) [74]
Yes	Metal, Polymer	Liquid Metal, Elastomer	vacuum casting, coating	-	Digital Light Processing (DLP)	Sequential	Separate	HAM	Characterization	Research	USA	(Deng et al., 2020) [75]
Yes	Polymer, Composite	Glass Fiber, Carbon Fiber, ABS	Hot Press Molding	-	Fused Deposition Modeling (FDM)	Sequential	Separate	HAM	Characterization	Research	India	(Dhandapani et al., 2023) [76]
No	Metal	Ti-6Al-4V		Milling	Powder Bed Fusion (PBF)	Sequential	Separate	HM	Characterization	Research	Israel	(Dolev et al., 2021) [77]
No	Metal	Ti-6Al-4V		Milling	Directed Laser Deposition (DLD)	Sequential	Separate	HM	Sustainability	Research	China	(Du et al., 2018) [29]
No	Metal	18Ni-300 Steel		Milling	Selective Laser Melting (SLM)	Sequential	Single	HM	Characterization	Research	China	(Du et al., 2018) [78]
No	Metal	316L SS	Dry EDM Milling (DEDM)		Plasma Arc Powder (PAP-WAAM)	Concurrent	Separate	HM	Characterization	Research	China	(Duan et al., 2023) [79]
No	Metal	AlSi5 Aluminum		Milling	Wire Arc Additive Manufacturing (WAAM)	Sequential	Separate	HM	Sustainability	Research	Slovenia	(Dugar et al., 2022) [80]
No	Ceramic	Silicon Carbide (SiC)	Structured Light Scanning	Machining	Binder Jetting (BJ)	Sequential	Separate	HM	Characterization	Research	USA	(Dvorak et al., 2023) [81]
No	Metal	Fe-25Al-1.5Ta		Hot Working (HW)	Laser Powder Bed Fusion (LPBF)	Sequential	Separate	HM	Characterization	Research	Germany	(Emdadi et al., 2023) [82]
-	-	-	Collaborative Assembly	-	-	Concurrent	In-situ	HHRM	Other	Robotics	The Netherlands	(Erasmus et al., 2018) [83]
No	Metal	316L SS		Machining	Directed Energy Deposition (DED)	Sequential	Single	HM	Characterization	Vehicle	USA	(Feldhausen et al., 2021) [39]
No	Metal	316L SS		Machining	Directed Energy Deposition (DED)	Sequential	Single	HM	Characterization	Research	USA	(Feldhausen et al., 2022) [84]
Yes	Metal	316L SS, 1060		Machining	Directed Energy Deposition (DED)	Sequential	Single	HM	Characterization	Research	USA	(Feldhausen et al., 2023) [85]
Yes	Polymer, Composite	PA12, Fiber-PA12		Machining	Fused Filament Fabrication (FFF)	Sequential	Separate	HM	Characterization	Research	Portugal	(Ferreira et al., 2020) [86]
Yes	Polymer	Resin, Medical Silicone GSM50	Casting	-	Stereolithography (SLA)	Manual	Separate	HAM	Education	Medicine	Australia	(Fitzgerald et al., 2023) [87]
Yes	Polymer, Composite	Kenaf, EVO Resin	autoclave	-	Vacuum-Assisted Resin Infusion (VARI)	Manual	Separate	HAM	Sustainability	Medicine	Mexico	(Franco-Urquiza et al., 2021) [88]
No	Metal	316L SS	single point incremental forming (SPIF)	-	Directed Energy Deposition (DED)	Sequential	Separate	HAM	Characterization	Research	Singapore	(Gao et al., 2022) [89]
No	Metal	S355J2	CMT	Machining	Wire Arc Additive Manufacturing (WAAM)	Sequential	Separate	HAM	Sustainability	Civil	Switzerland	(Ghafoori et al., 2023) [90]
No	Metal	316L SS		Wire-EDM (WEDM)	Powder Bed Fusion (PBF)	Sequential	Separate	HM	Characterization	Research	USA	(Gomez et al., 2021) [91]
No	Metal	316L SS		Machining	Directed Energy Deposition (DED)	Sequential	Single	HM	Characterization	Research	Ireland	(Gong et al., 2019) [92]
No	Polymer	ABS	Injection Molding (IM)	-	Fused Deposition Modeling (FDM)	Sequential	Separate	HAM	Other	Research	Ireland	(Gong et al., 2022) [93]
No	Polymer	PLA	Injection Molding (IM)	-	Fused Deposition Modeling (FDM)	Sequential	Separate	HAM	Characterization	Research	China	(Gong et al., 2022) [94]
Yes	Metal	Hastelloy, Inconel 718		Machining	Directed Energy Deposition (DED)	Sequential	Separate	HM	Sustainability	Research	Spain	(Gonzalez-Barrio et al., 2022) [95]
Yes	Metal, Polymer	PBT, Silver Paste		-	ARBURG Plastic Freeforming (APF)	Sequential	Separate	HAM	Characterization	Electronics	Germany	(Granse et al., 2023) [96]
No	Metal	316L SS	High-speed laser directed energy deposition (HS L-DED)	Micro Milling	Laser Powder Bed Fusion (LPBF)	Sequential	Separate	HM	Characterization	Micromanufacturing	Germany	(Greco et al., 2022) [97]
No	Metal	Inconel 738LC		Machining	Selective Laser Melting (SLM)	Sequential	Separate	HM	Characterization	Research	China	(Guo et al., 2021) [98]
No	Metal	18Ni300 Maraging Steel		Milling	Selective Laser Melting (SLM)	Sequential	Separate	HM	Characterization	Research	China	(Guo et al., 2023) [99]
No	Metal	1070 Steel	Manual Stacking	-	Shot Peening (SP)	Manual	Separate	HAM	Characterization	Vehicle	USA	(Hadidi et al., 2020) [100]
Yes	Metal	Ti6Al4V, NiTi SMA	Purchased	-	Laser Powder Bed Fusion (LPBF)	Sequential	Separate	HAM	Characterization	Medicine	Germany	(Hamann et al., 2021) [101]
No	Metal	316L SS		Machining	Laser Metal Deposition (LMD)	Sequential	Separate	HM	Sustainability	Research	China	(He et al., 2020) [102]
No	Metal	Structural Steel	Layer laminated manufacturing (LLM) – LOM	-	LOM	Sequential	Separate	HM	Optimization	Research	Germany	(Helfesrieder et al., 2022) [103]
No	Metal	Ti-6Al-4V	Hot Forging	-	Laser Directed Energy Deposition (L-DED)	Sequential	Separate	HAM	Characterization	Research	Germany	(Hemes et al., 2021) [104]
Yes	Metal, Ceramic	Copper, SiC		Etching	Electroplating	Sequential	Separate	HM	Characterization	Electronics	USA	(Herrault et al., 2020) [105]
No	Metal	Titanium Alloy (Ti Alloy)		Milling	Laser Hotwire Directed Energy Deposition (DED)	Sequential	Separate	HM	Characterization	Research	USA	(Honeycutt et al., 2021) [106]
Yes	Metal, Ceramic, Polymer	PMMA, Silver Nanoparticles, TiO_2_	Focused Ion Beam (FIB) Milling	Micro Milling	Aerodynamically Focused Nanoparticles Printing (AFN)	Sequential	Separate	HM	Characterization	Micromanufacturing	South Korea	(Jang et al., 2020) [107]
No	Polymer	PLA		Machining	Fused Deposition Modeling (FDM)	Sequential	Separate	HM	Characterization	Research	South Korea	(Lee et al., 2018) [108]
Yes	Metal	Nano-AlSi10Mg + Al_2_O_3_	Casting	Machining	Binder Jetting (BJ)	Sequential	Separate	HM	Characterization	Research	Germany	(Lee et al., 2021) [109]
No	Metal	304 SS	Ultrasonic vibration-assisted laser polishing (UVLP)	-	-	Assisted	Separate	HSM	Characterization	Research	China	(Lee et al., 2022) [110]
No	Metal	316L SS		Wire-EDM (WEDM)	Hot Wire Deposition (HWD)	Sequential	Separate	HM	Characterization	Research	USA	(Kaiser et al., 2023) [111]
No	Ceramic	Sapphire	micromilling	Laser Machining (LM)	-	Sequential	Separate	HSM	Characterization	Micromanufacturing	Japan	(Kang et al., 2020) [112]
No	Metal	316L SS		Machining	Selective Laser Melting (SLM)	Sequential	Separate	HM	Characterization	Medicine	Turkey	(Kannan et al., 2021) [113]
No	Metal	Ti Alloy	milling	Laser Ablation (LA)	-	Manual	Separate	HSM	Characterization	Medicine	Poland	(Katahira et al., 2020) [114]
Yes	Composite	Acrylic Mixture	UV Curing	-	Paste Extrusion	Assisted	Separate	HM	Characterization	Medicine	Finland	(Kaynak et al., 2018) [115]
No	Polymer	PDMS	Laser-based machining	Micro-Drilling (MD)	-	Sequential	Separate	HSM	Characterization	Medicine	USA	(Komorowski et al., 2020) [116]
No	Metal	Copper (Cu)	Focused ion beam	Micro-Electrical Discharge Machining (MEDM)	-	Sequential	Separate	HSM	Characterization	Micromanufacturing	South Korea	(Kretzschmar et al., 2019) [117]
Yes	Metal	316L, 40 Steel		Machining	Directed Energy Deposition (DED)	Sequential	Single	HM	Characterization	Research	China	(Li et al., 2018) [118]
No	Metal	Al 6061		Milling	Bending	Sequential	Separate	HM	Characterization	Research	USA	(Li et al., 2018) [119]
No	Metal	316L SS Powder, Inconel 718 Powder		Thermal Milling (TM)	Directed Laser Deposition (DLD)	Sequential	Separate	HM	Characterization	Research	China	(Li et al., 2019) [120]
Yes	Metal	Inconel 718 Powder, 316L SS		Thermal Milling (TM)	Directed Energy Deposition (DED)	Sequential	Separate	HM	Characterization	Research	China	(Li et al., 2019) [121]
Yes	Metal	Q235, H08Mn2Si		Groove Machining (GM)	Wire Arc Additive Manufacturing (WAAM)	Sequential	Separate	HM	Sustainability	Research	China	(Li et al., 2019) [122]
No	Metal	Ti-6Al-4V		Milling	Direct Material Deposition (DMD)	Sequential	Separate	HM	Characterization	Research	China	(Li et al., 2020) [31]
No	Metal	5052 Al	Heat (air heat gun)	-	Bending	Assisted	Single	HM	Characterization	Research	China	(Li et al., 2020) [123]
No	Metal	316L SS		Milling	Directed Energy Deposition (DED)	Sequential	Single	HM	Characterization	Research	China	(Li et al., 2021) [124]
No	Metal	316L SS		Thermal Milling (TM)	Directed Energy Deposition (DED)	Sequential	Separate	HM	Characterization	Research	USA	(Li et al., 2021) [125]
No	Metal	Ti-6Al-4V	Electrochemical Machining (ECM)		Laser Cladding Deposition	Sequential	Separate	HM	Characterization	Research	USA	(Li et al., 2022) [126]
Yes	Metal, Polymer	PDMS, Resin, Cu	Surface Metallization	-	Stereolithography (SLA)	Sequential	Separate	HAM	Characterization	Research	China	(Li et al., 2022) [127]
Yes	Composite	Graphene-Metal	Laser Shock Peening (LSP)		Selective Laser Sintering (SLS)	Sequential	Separate	HAM	Characterization	Research	USA	(Lin et al., 2018) [128]
No	Polymer	Silicone (Polysiloxane)	Material Extrusion	-	Binder Jetting (BJ)	Sequential	Single	HAM	Characterization	Medicine	Canada	(Liravi et al., 2018) [129]
No	Polymer	Silicone	Material Extrusion	-	Material Jetting (MJ)	Sequential	Single	HAM	Characterization	Medicine	Canada	(Liravi et al., 2018) [130]
No	Metal	316L SS	AECJM	-	-	Assisted	Single	HSM	Characterization	Micromanufacturing	China	(Liu et al., 2018) [131]
No	Metal	AlMg5Cr		Milling	Arc Welding	Sequential	In-situ	HM	Characterization	Research	China	(Liu et al., 2020) [132]
Yes	Metal	2024-T3, Fe316L	Incremental Forming (ISF)	-	Thermal Spraying	Manual	Separate	HAM	Characterization	Research	China	(Liu et al., 2021) [133]
No	Metal	316L Powder		Milling	Metal Laser Direct Deposition (MLDD)	Sequential	Single	HM	Energy Consumption	Vehicle	China	(Liu et al., 2021) [134]
No	Polymer	PETG	FSW (Friction Stir Welding)	-	Extrusion-Based Additive Manufacturing (EAM)	Sequential	Separate	HAM	Characterization	Research	China	(Liu et al., 2023) [135]
Yes	Polymer	PVC, PLA, PVA		Milling	Fused Filament Fabrication (FFF)	Sequential	Separate	HM	Characterization	Civil	Italy	(Liverani et al., 2023) [136]
No	Metal	Ti-6Al-4V		Milling	Laser Powder Bed Fusion (LPBF)	Sequential	Separate	HM	Characterization	Vehicle	Spain	(Loyda et al., 2023) [137]
No	Metal	Ti-6Al-4V	Forging	-	Laser Metal Deposition (LMD)	Sequential	Separate	HAM	Characterization	Research	China	(Ma et al., 2021) [138]
Yes	Metal	SS Corrax, PH13-8Mo		Machining	Selective Laser Melting (SLM)	Sequential	Separate	HM	Characterization	Research	Spain	(Marin et al., 2021) [139]
Yes	Metal	SS Corrax, PH13-8Mo		Machining	Laser Powder Bed Fusion (LPBF)	Sequential	Separate	HM	Characterization	Research	Spain	(Marin et al., 2023) [140]
No	Metal	316L SS		Milling	WAAM, Rolling, LPC	Sequential	Separate	HM	Characterization	Research	Czech Republic	(Masek et al., 2019) [141]
No	Metal	316L SS	PBF (Powder Bed Fusion)	-	Metal Injection Moulding (MIM)	Sequential	Separate	HAM	Characterization	Research	UK	(Mehmeti et al., 2020) [142]
No	Metal	316L SS	Hot Isostatic Pressing (HIP)	-	MIM, PBF	Sequential	Separate	HAM	Characterization	Research	UK	(Mehmeti et al., 2021) [143]
Yes	Metal	CM247LC, Inconel 718		Machining	Laser-Directed Energy Deposition (L-DED)	Sequential	Single	HM	Sustainability	Vehicle	UK	(Mehmeti et al., 2022) [144]
Yes	Polymer	PCL, Gelatin, HFIP, GA	Electrospinning, Freeze-Casting	-	Selective Laser Sintering (SLS)	Sequential	Separate	HAM	Characterization	Medicine	China	(Meng et al., 2023) [145]
Yes	Composite	Stamp Sand, ASA	Injection Molding	-	Fused Deposition Modeling (FDM)	Sequential	Separate	HAM	Sustainability	Research	USA	(Meyer et al., 2020) [146]
No	Metal	Ti-6Al-4V	Forging	-	Wire Arc Additive Manufacturing (WAAM)	Sequential	Separate	HAM	Characterization	Vehicle	Germany	(Mishurova et al., 2020) [147]
Yes	Polymer	PLA, ABS	Chemical immersion in acetone	-	Material Treatment Extrusion Additive Manufacturing (MaTrEx AM)	Sequential	Separate	HAM	Characterization	Research	UK	(Moetazedian et al., 2021) [148]
No	Metal	AlMg5		Turning (T)	Laser Metal Wire Deposition (LMWD)	Sequential	Separate	HM	Characterization	Vehicle	Germany	(Mohring et al., 2022) [149]
No	Metal	Ti-6Al-4V		Cryogenic Milling	Laser Metal Deposition (LMD)	Concurrent	In-situ	HM	Characterization	Vehicle	Germany	(Moritz et al., 2020) [25]
No	Metal	22MnB5		Milling	Selective Laser Melting (SLM)	Sequential	Separate	HM	Characterization	Research	South Africa	(Muvunzi et al., 2020) [150]
Yes	Metal	18Ni-300, 4140-steel	Heat Treatment	Machining	3D Printing	Cyclical	Single	HM	Characterization	Research	Canada	(Osman et al., 2023) [151]
No	Metal	Inconel 718		Machining	Laser Metal Deposition (LMD)	Sequential	Separate	HM	Characterization	Research	Spain	(Ostra et al., 2019) [28]
No	Metal	316L SS		Milling	Extrusion Freeform Fabrication (EFF)	Sequential	Separate	HM	Characterization	Research	Italy	(Parenti et al., 2018) [152]
No	Polymer	PLA		Milling	Fused Deposition Modeling (FDM)	Sequential	Separate	HM	Optimization	Research	Romania	(Pascu et al., 2023) [153]
No	Polymer	PGS-M		Milling	Material Jetting (MJ)	Sequential	Separate	HAM	Characterization	Medicine	UK	(Pashneh-Tala et al., 2020) [154]
No	Polymer	PLA		Machining	Material Extrusion	Sequential	Separate	HM	Characterization	Research	Spain	(Paz et al., 2018) [155]
No	Metal	Inconel 718		Milling	Laser Powder Bed Fusion (LPBF)	Sequential	Separate	HM	Characterization	Research	Spain	(Perez-Ruiz et al., 2021) [156]
No	Metal	HSS M3:2, HWS H11	Heat	Micromilling (MML)	-	Assisted	Single	HSM	Characterization	Micromanufacturing	Germany	(Platt et al., 2020) [157]
Yes	Polymer	PLA, Silicone	Overmolding	-	Fused Deposition Modeling (FDM)	Sequential	Separate	HAM	Other	Medicine	Romania	(Popescu et al., 2020) [158]
No	Metal	316L SS	Polishing	Wire-EDM (WEDM	Selective Laser Melting (SLM)	Sequential	Separate	HM	Other	Research	Portugal	(Pragana et al., 2020) [159]
No	Metal	Ti-6Al-4V	SLM	-	Laser Deposition Manufacturing (LDM)	Sequential	Separate	HAM	Characterization	Research	China	(Qin et al., 2019) [160]
No	Metal	Metal	High-Speed Machining (HSM)		SLM, LMD, WAAM	Sequential	Separate	HM	Other	Vehicle	France	(Rauch et al., 2022) [161]
No	Metal	Inconel 738LC	High-Speed Milling (HSM)		Selective Laser Melting (SLM)	Sequential	Separate	HM	Characterization	Research	China	(Ren et al., 2023) [162]
Yes	Metal	TiAl, Ti-Nb-Mo	Casting, LPBF	-	Directed Energy Deposition (DED)	Sequential	Separate	HAM	Sustainability	Vehicle	Germany	(Rittinghaus et al., 2020) [163]
Yes	Metal, Polymer	Silver Ink, ABS	Microdispensing	-	Fused Filament Fabrication (FFF)	Sequential	Single	HAM	Characterization	Electronics	USA	(Robles et al., 2019) [164]
Yes	Polymer	Vero Mixture	Deposition	-	Polyjet	Sequential	Separate	HAM	Characterization	Medicine	USA	(Ruiz et al., 2020) [165]
-	-	-	Collaborative Assembly	-	-	Concurrent	In-situ	HHRM	Other	Robotics	USA	(Sadrfaridpour et al., 2018) [166]
No	Metal	MgCa0.8	finish burnishing	Hybrid Dry Cutting (HDC)	-	Sequential	Separate	HSM	Characterization	Medicine	USA	(Salahshoor et al., 2018) [167]
No	Metal	18Ni-300 Maraging Steel	High-Speed Machining (HSM)		Laser Powder Bed Fusion (LPBF)	Cyclical	Single	HM	Characterization	Research	Canada	(Sarafan et al., 2021) [168]
No	Metal	316L SS	High-Speed Machining (HSM)		Laser Powder Bed Fusion (LPBF)	Cyclical	Single	HM	Characterization	Research	Canada	(Sarafan et al., 2022) [169]
No	Metal	Mg WE43	Interlayer Ultrasonic Peening	-	Additive Manufacturing (AM)	Sequential	Separate	HM	Characterization	Research	USA	(Sealy et al., 2021) [170]
-	-	-		Milling	Wire Arc Additive Manufacturing (WAAM)	Sequential	Separate	HM	Optimization	Research	China	(Shen et al., 2021) [171]
No	Metal	Al-Cu		Laser Welding (LW)	Wire Arc Additive Manufacturing (WAAM)	Sequential	Separate	HAM	Characterization	Vehicle	China	(Shi et al., 2023) [172]
Yes	Metal	AlSi10Mg, AW-6082		Machining	Selective Laser Melting (SLM)	Sequential	Separate	HAM	Sustainability	Research	Portugal	(Silva et al., 2022) [173]
No	Metal	Inconel 718	DMD	Milling	Investment Casting	Sequential	Separate	HM	Sustainability	Research	Switzerland	(Soffel et al., 2021) [174]
No	Metal	Maraging Steel	High-Speed Milling (HSM)		Selective Laser Melting (SLM)	Cyclical	Single	HM	Characterization	Micromanufacturing	Germany	(Sommer et al., 2021) [175]
Yes	Metal, Polymer	Copper, Silver, PET		Laser Ablation (LA)	Nanoparticle Deposition System (NPDS)	Sequential	Single	HAM	Characterization	Electronics	South Korea	(Song et al., 2020) [176]
Yes	Metal	Copper, Aluminum	physical vapor deposition	-	Stereolithography (SLA)	Sequential	Separate	HAM	Characterization	Electronics	Poland	(Sorocki et al., 2020) [177]
No	Metal	316L SS		Milling	Laser Deposition (DLM)	Sequential	Separate	HM	Sustainability	Vehicle	Greece	(Stavropoulos et al., 2020) [178]
No	Metal	GH4169 (Inconel 718)	laser shock processing (LSP), deep cryogenic treatment (DCT)	Machining	-	Sequential	Separate	HM	Characterization	Research	China	(Sun et al., 2019) [179]
No	Metal	Inconel 625		Milling	Directed Energy Deposition (DED)	Sequential	Separate	HM	Characterization	Research	USA	(Sunny et al., 2021) [180]
No	Metal	Mild Steel, SG3, G4Si	Forming	-	Wire Arc Additive Manufacturing (WAAM)	Sequential	Separate	HAM	Characterization	Research	Germany	(Sydow et al., 2022) [181]
No	Metal	316L SS		Machining	Directed Energy Deposition (DED)	Sequential	Separate	HM	Characterization	Research	UK	(Tapoglou et al., 2021) [38]
Yes	Metal	15-5 PH SS, Inconel 718, Ti-6Al-4V		Machining	Directed Energy Deposition (DED)	Sequential	Separate	HM	Characterization	Research	UK	(Tapoglou et al., 2022) [182]
Yes	Polymer	PVA, TPU, PA	Selective Laser Sintering (SLS)	-	Fused Filament Fabrication (FFF)	Sequential	Separate	HAM	Education	Medicine	Spain	(Tejo-Otero et al., 2021) [183]
No	Metal	ER316LSi		Machining	Directed Energy Deposition (DED)	Sequential	Single	HM	Optimization	Research	USA	(Thien et al., 2021) [184]
No	Metal	2219 Al	Heat	Thermally Assisted Machining (TAM)	Wire Arc Additive Manufacturing (WAAM)	Assisted	In-situ	HM	Characterization	Research	China	(Tian et al., 2022) [185]
Yes	Ceramic, Composite	Silicon, MMCs	soft tooling fabrication, slurry casting, debinding-sintering, metal infiltration	-	-	Sequential	Separate	HM	Characterization	Research	USA	(Togwe et al., 2020) [186]
Yes	Metal	AlSi10Mg, A356-T6	casting, forging	Machining	Selective Laser Sintering (SLS)	Sequential	Separate	HM	Characterization	Vehicle	Italy	(Tommasi et al., 2021) [187]
No	Metal	Co-Cr Alloy		Milling	Repeated Laser Sintering (RLS)	Sequential	Separate	HM	Characterization	Medicine	Japan	(Torii et al., 2018) [188]
-	-	-	Collaborative Assembly	-	-	Concurrent	In-situ	HHRM	Characterization	Robotics	The Netherlands	(Traganos et al., 2021) [189]
No	Metal	420 SS		Machining	Laser Engineered Net Shaping (LENS)	Sequential	Separate	HM	Characterization	Research	Canada	(Urbanic et al., 2018) [190]
No	Polymer	ABS		Machining	Fused Deposition Modeling (FDM)	Sequential	Separate	HM	Cost	Research	Canada	(Urbanic et al., 2019) [191]
No	Metal	316L SS		Machining	Directed Energy Deposition (DED)	Sequential	Separate	HM	Characterization	Research	USA	(Vaughan et al., 2022) [192]
No	Polymer	Poly (L-Lactic Acid) (PLA)	Electrospinning	-	Fused Deposition Modeling (FDM)	Sequential	Separate	HAM	Characterization	Medicine	Mexico	(Vazquez-Armendariz et al., 2020) [193]
No	Metal	Ti-6Al-4V		Machining	Wire Arc Additive Manufacturing (WAAM)	Sequential	Separate	HM	Characterization	Vehicle	Spain	(Veiga et al., 2020) [194]
Yes	Metal	CM247LC, IN718	Hot Isostatic Pressing		Selective Laser Melting (SLM)	Sequential	Separate	HAM	Characterization	Vehicle	UK	(Wang et al., 2020) [195]
Yes	Composite	Resin, Carbon	Chemical Activation, Pyrolysis	-	Stereolithography (SLA)	Sequential	Separate	HAM	Characterization	Research	UK	(Wang et al., 2020) [196]
Yes	Metal	Magnesium, Zinc, Calcium	Hot Extrusion Process	Turning Induced Deformation Technique (TID)	Disintegrated Melt Deposition (DMD)	Sequential	Separate	HM	Sustainability	Research	Singapore	(Wang et al., 2022) [197]
Yes	Metal	AlSi10Mg, Al6061	Forging	-	Selective Laser Melting (SLM)	Sequential	Separate	HAM	Characterization	Research	China	(Wang et al., 2022) [198]
Yes	Metal, Polymer	PLA, ABS, Silver Ink	Paste Extrusion, Pick and Place	-	Fused Filament Fabrication (FFF)	Sequential	Single	HAM	Toolpath	Electronics	Germany	(Wasserfall et al., 2020) [199]
Yes	Polymer	Resin, Silicone	Silicone Casting	-	Stereolithography (SLA)	Sequential	Separate	HAM	Education	Medicine	Australia	(Weatherall et al., 2021) [200]
Yes	Polymer, Composite	PDMS, Graphene Ink	Aerosol Jet Deposition	-	Electro-hydrodynamic Jet (E-jet) Printing	Sequential	Separate	HAM	Characterization	Electronics	UK	(Wilkinson et al., 2020) [201]
Yes	Polymer	Polymer Fiber, Hydrogel		-	Electrospinning	Assisted	Single	HAM	Characterization	Medicine	USA	(Williams et al., 2018) [202]
No	Metal	Stainless Steel		Milling	Directed Energy Deposition (DED)	Sequential	Single	HM	Energy Consumption	Research	USA	(Wippermann et al., 2020) [203]
Yes	Metal, Composite	CNC, GNP, Copper	Vacuum Filtration, IPL Sintering, Mechanical Hot Pressing, Heat Treatment	-	-	Sequential	Separate	HM	Characterization	Research	Canada	(Wong et al., 2020) [204]
No	Metal	316L SS		Milling	Laser Metal Deposition (LMD)	Sequential	Separate	HM	Characterization	Research	China	(Wu et al., 2021) [205]
No	Polymer	ma-P1275G		Laser Direct Write Lithography (DWL)	Nanoimprint Lithography (NIL)	Sequential	Separate	HM	Characterization	Micromanufacturing	Switzerland	(Xie et al., 2021) [206]
Yes	Polymer	PCL Mixture	Fused Filament Fabrication (Overprinting), Hot Melt Extrusion	-	Injection Molding	Sequential	Separate	HAM	Characterization	Medicine	Ireland	(Xu et al., 2023) [207]
No	Metal	Ti-6Al-4V		Milling	Laser Metal Deposition (LMD)	Sequential	Separate	HM	Characterization	Research	USA	(Yan et al., 2018) [208]
No	Metal	316L SS		Milling	Directed Energy Deposition (DED)	Sequential	Single	HM	Characterization	Research	China	(Yang et al., 2018) [209]
No	Metal	316L SS		Machining	Directed Energy Deposition (DED)	Sequential	Single	HM	Characterization	Research	China	(Yang et al., 2021) [210]
-	-	-		-	-	Sequential	Single	HHRM	Toolpath	Robotics	China	(Zeng et al., 2018) [211]
No	Metal	Al5Si Aluminum		Milling	Wire Arc Additive Manufacturing (WAAM)	Sequential	Separate	HM	Characterization	Research	China	(Zhang et al., 2019) [212]
No	Metal	316L SS		Machining	Directed Energy Deposition (DED)	Sequential	Single	HM	Toolpath	Research	USA	(Zhang et al., 2020) [213]
No	Metal	Al5Si Aluminum		Milling	Wire Arc Additive Manufacturing (WAAM)	Sequential	Separate	HM	Characterization	Research	China	(Zhang et al., 2021) [214]
-	-	-		Machining	Powder Bed Fusion (PBF)	Sequential	Separate	HM	Cost	Research	Canada	(Zheng et al., 2020) [215]
No	Metal	Ti-6Al-4V		-	Investment Casting	Sequential	Separate	HAM	Characterization	Research	China	(Zong et al., 2023) [216]

## Data Availability

No new data were created or analyzed in this study. Data sharing is not applicable to this article.
